# Probing conformational transitions towards mutagenic Watson–Crick-like G·T mismatches using off-resonance sugar carbon *R*_1ρ_ relaxation dispersion

**DOI:** 10.1007/s10858-020-00337-7

**Published:** 2020-08-12

**Authors:** Atul Rangadurai, Eric S. Szymanski, Isaac Kimsey, Honglue Shi, Hashim M. Al-Hashimi

**Affiliations:** 1 Department of Biochemistry, Duke University School of Medicine, Durham, NC 27710 USA; 2 Present Address: Nymirum, 4324 S. Alston Avenue, Durham, NC 27713 USA; 3 Department of Chemistry, Duke University, Durham, NC 27710 USA

**Keywords:** Tautomers, Anions, Replication error, Nucleic acid dynamics, Chemical exchange, Sugar pucker

## Abstract

NMR off-resonance *R*_1ρ_ relaxation dispersion measurements on base carbon and nitrogen nuclei have revealed that wobble G·T/U mismatches in DNA and RNA duplexes exist in dynamic equilibrium with short-lived, low-abundance, and mutagenic Watson–Crick-like conformations. As Watson–Crick-like G·T mismatches have base pairing geometries similar to Watson–Crick base pairs, we hypothesized that they would mimic Watson–Crick base pairs with respect to the sugar-backbone conformation as well. Using off-resonance *R*_1ρ_ measurements targeting the sugar C3′ and C4′ nuclei, a structure survey, and molecular dynamics simulations, we show that wobble G·T mismatches adopt sugar-backbone conformations that deviate from the canonical Watson–Crick conformation and that transitions toward tautomeric and anionic Watson–Crick-like G·T mismatches restore the canonical Watson–Crick sugar-backbone. These measurements also reveal kinetic isotope effects for tautomerization in D_2_O versus H_2_O, which provide experimental evidence in support of a transition state involving proton transfer. The results provide additional evidence in support of mutagenic Watson–Crick-like G·T mismatches, help rule out alternative inverted wobble conformations in the case of anionic G·T^−^, and also establish sugar carbons as new non-exchangeable probes of this exchange process.

**Electronic supplementary material:**

The online version of this article (10.1007/s10858-020-00337-7) contains supplementary material, which is available to authorized users.

## Introduction

In their paper describing the structure of the DNA double helix, James Watson and Francis Crick proposed that spontaneous mutations could arise when nucleotide bases adopt rare and energetically less favorable tautomeric forms as this could allow wobble mismatches such as A·C and G·T to adopt a geometry similar to that of canonical G-C and A-T Watson–Crick (WC) base pairs (bps) (Watson and Crick 1953). Two decades later, Topal and Fresco extended this idea, and proposed that the probability with which rare tautomers form could determine the probability of misincorporating mismatches during replication and translation (Topal and Fresco 1976; Topal and Fresco 1976. During the same period, it was shown that WC-like G·T mismatches could also form via ionization of the thymine base Lawley and Brookes 1962; Sowers et al. 1987; Lawley and Brookes 1961. This anionic WC-like species could explain the increase in G·T misincorporation probability with increasing pH. Yu et al. (1993) Together, the tautomeric and anionic WC-like G·T mismatches could rationalize why chemical modifications that stabilize or mimic either tautomeric (Harris et al. 2003; Budowsky 1976; Woodside and Guengerich 2002; Cantara et al. 2013; Vendeix et al. 2012; Kurata et al. 2008; Ikeuchi et al. 2010) or ionic (Yu et al. 1993; Strebitzer et al. 2018; Weixlbaumer et al. 2007) forms of the bases increase the probability of errors during replication and translation.

**Fig. 1:**
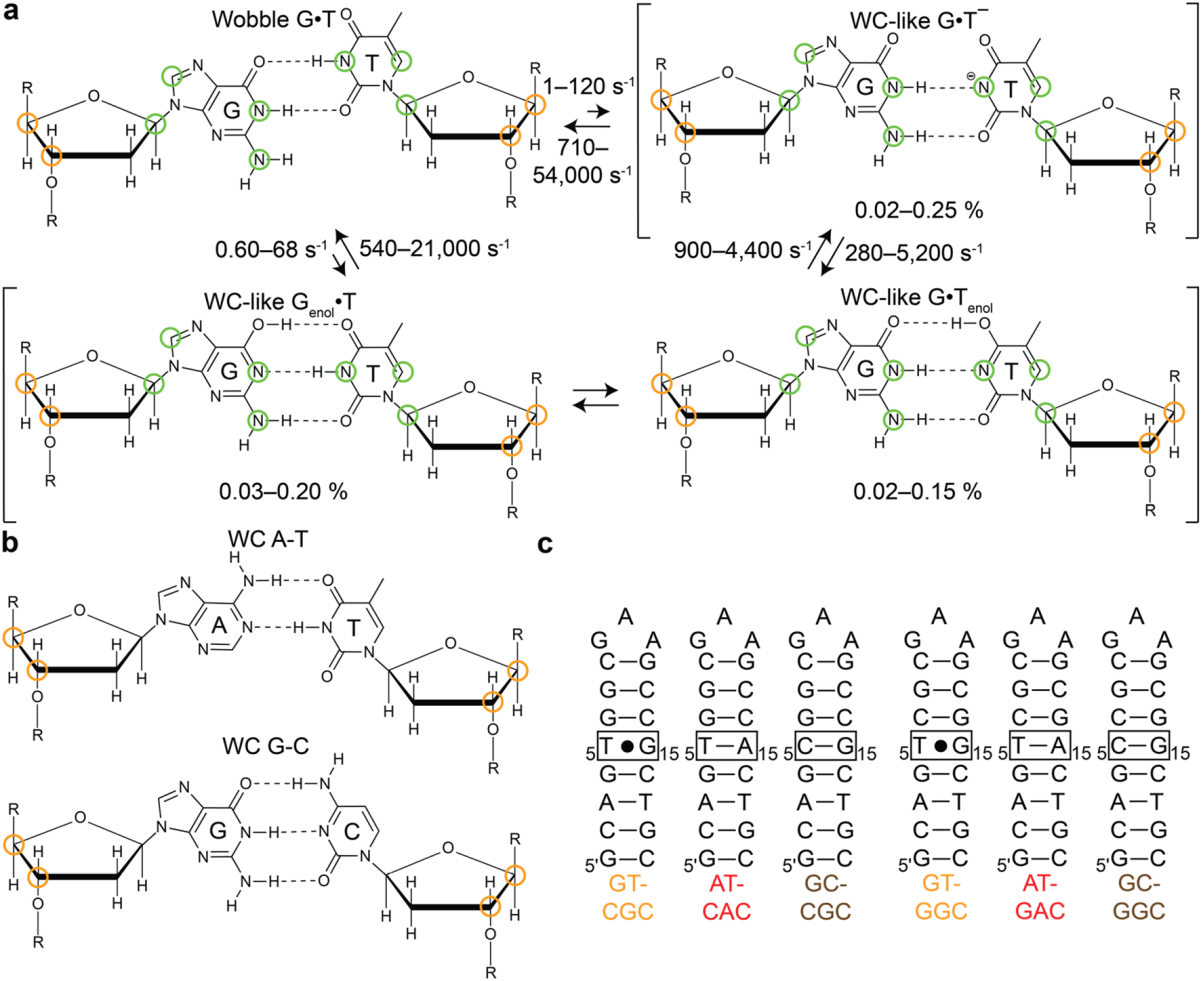
**a** Exchange between wobble and, tautomeric and anionic WC-like G·T mismatches^29^. Green circles denote RD probes used to characterize the exchange in previous studies (Szymanski et al. 2017; Kimsey et al. 2015). **b** A-T and G-C Watson–Crick bps in DNA. Orange circles denote RD probes used to characterize formation of WC-like mismatches in this study. **c** DNA constructs used in this study

Studies over the past decade have provided additional structural and kinetic evidence in support of WC-like mismatches having important roles in the generation of errors during replication and translation. WC-like mismatches have been observed in crystal structures of DNA polymerases (Wang et al. 2011; Bebenek et al. 2011; Koag et al. 2014; Koag and Lee 2018; Sharma et al. 2013) and the ribosome (Rozov et al. 2018; Rozov et al. 2016; Rozov et al. 2015; Demeshkina et al. 2012) in catalytically active conformations. Spontaneous transitions between wobble G·T/U mismatches and short-lived, low-abundance WC-like tautomeric and anionic G·T/U mismatches have also been observed in solution using off-resonance *R*_1ρ_ NMR relaxation dispersion (RD) experiments (Rangadurai et al. 2019; Palmer and Massi 2006) in both DNA and RNA duplexes (Fig. 1a). Szymanski et al. (2017), Kimsey et al. (2018) and Kimsey et al. (2015)) WC-like G·T mismatches were shown to form with probabilities that could quantitatively explain the ~ 1000 fold reduction in misincorporation rate for G·T mismatches compared to G-C bps (Kimsey et al. 2018. Beyond their roles in the generation of errors during replication and translation, WC-like mismatches have also been proposed to play roles in the generation of errors during transcription (Phillips and Brown 1967; Singer and Spengler 1981; Li et al. 2014) and in DNA damage repair (Freudenthal et al. 2015).

Dynamic transitions between wobble and WC-like G·T/U mismatches (Fig. 1a) (Szymanski et al. 2017; Kimsey et al. 2018; Kimsey et al. 2015) have so far been studied by measuring off-resonance *R*_1ρ_ NMR RD data for nitrogen and carbon spins in the nucleotide bases. These include the imino nitrogens (G-N1 and T/U-N3), which experience large downfield shifts (~ 50 ppm) upon deprotonation, as well as the aromatic carbons (G-C8 and T-C6) and amino nitrogen (G-N2) atoms, which experience smaller downfield shifts (~ 2–4 ppm). These RD measurements (Kimsey et al. 2018; Kimsey et al. 2015) combined with single atom substitution experiments have identified three distinct WC-like G·T/U mismatches stabilized by WC-like hydrogen-bonds (Szymanski et al. 2017). These include two rapidly interconverting tautomeric forms in which either the G or T/U base can tautomerize (G^enol^ and T^enol^/U^enol^) to form tautomeric WC-like G^enol^·T/U and G·T^enol^/U^enol^ mismatches, and an anionic species in which the T/U ionizes to form anionic WC-like G·T^−^/U^−^_-_mismatches. However, it remains unknown whether these transitions are also accompanied by changes in the sugar-backbone conformation.

Compared to WC bps, G·T wobble mismatches adopt non-canonical sugar-backbone conformations (Allawi and SantaLucia 1998; Patel et al. 1982; Hare et al. 1986; Hunter et al. 1987). If the transient low-abundance species measured by NMR RD does indeed represent a WC-like G·T mismatch, one would expect that such a transition might restore a canonical WC-like sugar-backbone conformation (Fig. 1b). In principle, one can probe such transient conformational changes in the sugar-backbone using RD measurements targeting the sugar carbons C3′ and C4′ (Shi et al. 2018; Clay et al. 2017). Beyond confirming the identity of the transient low-abundance species measured previously by RD and establishing sugar RD as a new probe of this exchange process, determining the sugar-backbone conformation of Watson–Crick like G·T mismatches is important for assessing their mutagenicity as it defines the placement of the phosphate oxygen atom, which is integral for polymerization chemistry during replication.

Here, we used off-resonance sugar carbon *R*_1ρ_ measurements, a structure survey, and computational Molecular Dynamics (MD) simulations to characterize changes in the sugar-backbone accompanying the formation of tautomeric and anionic WC-like G·T mismatches. The results show that the G·T wobble mismatch adopts a non WC-like sugar-backbone conformation and that transitions toward either tautomeric or anionic WC-like G·T mismatches restore the canonical WC-like sugar-backbone conformation. These measurements extend the definition of a WC-like bp to encompass the sugar and backbone, help rule out alternative inverted wobble conformations in the case of anionic G·T^–^, and also offer new non-exchangeable probes of this exchange process that can enable measurements over a broad range of structural environments.

## Materials and methods

### Crystal structure survey of G·T mismatches

**Fig. 2:**
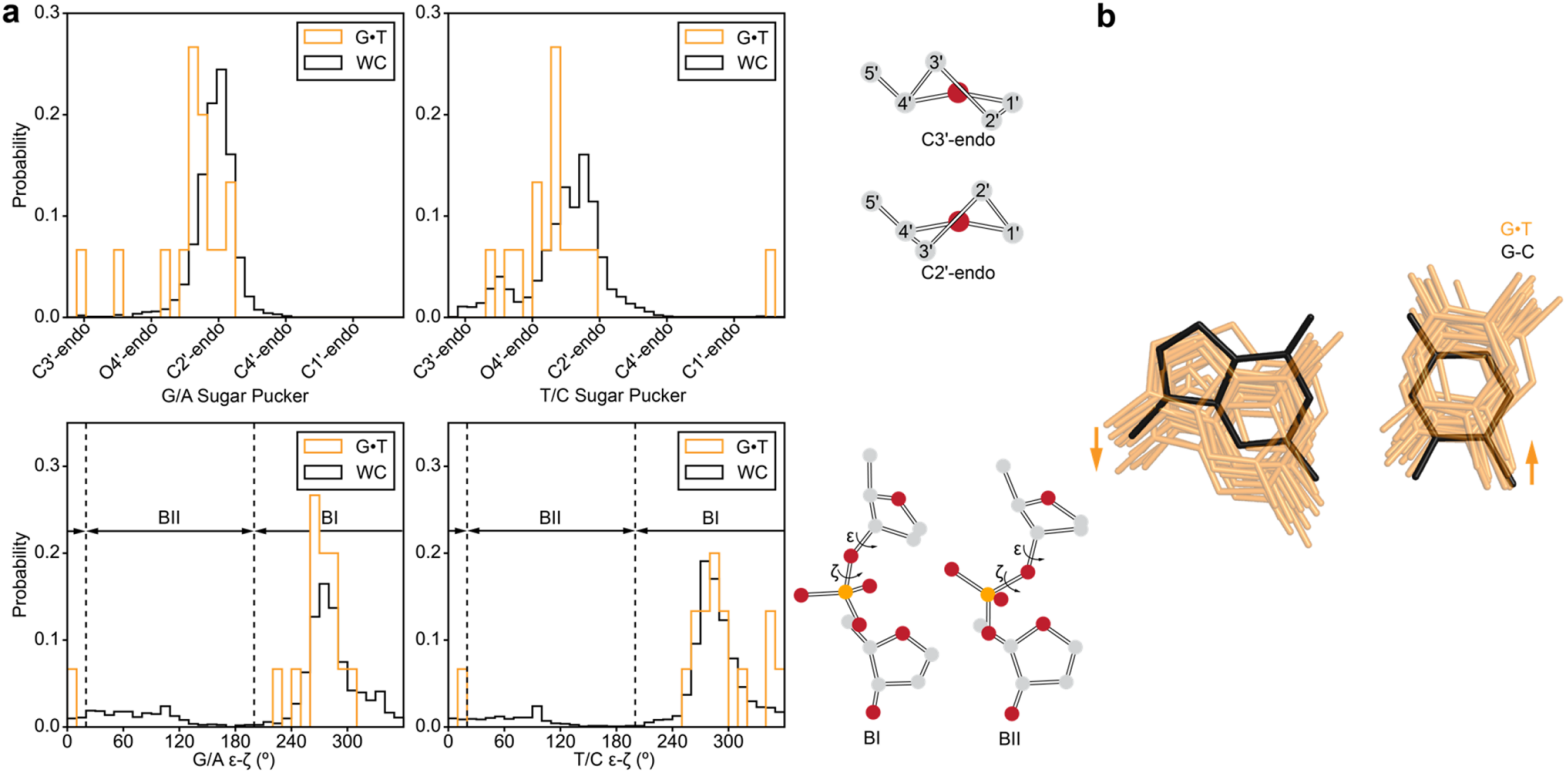
**a** Histograms comparing the sugar pucker and ε−ζ torsion angle for G·T mismatches (orange) and WC bps (black), as obtained from a survey of crystal structures in the PDB^41^ (Materials and Methods). The angles for G and T in the G·T mismatch were compared to those of G/A and T/C in the WC bps, respectively. The C2′-endo and C3′-endo sugar puckers as well as BI and BII backbone conformations are shown on the right. **b** Overlay of crystal structures of G·T mismatches obtained from a survey of the PDB (orange) and an idealized G-C bp (black) (Materials and Methods). Arrows indicate direction of movement of bases required for transition from a WC to a wobble bp conformation

**Fig. 3:**
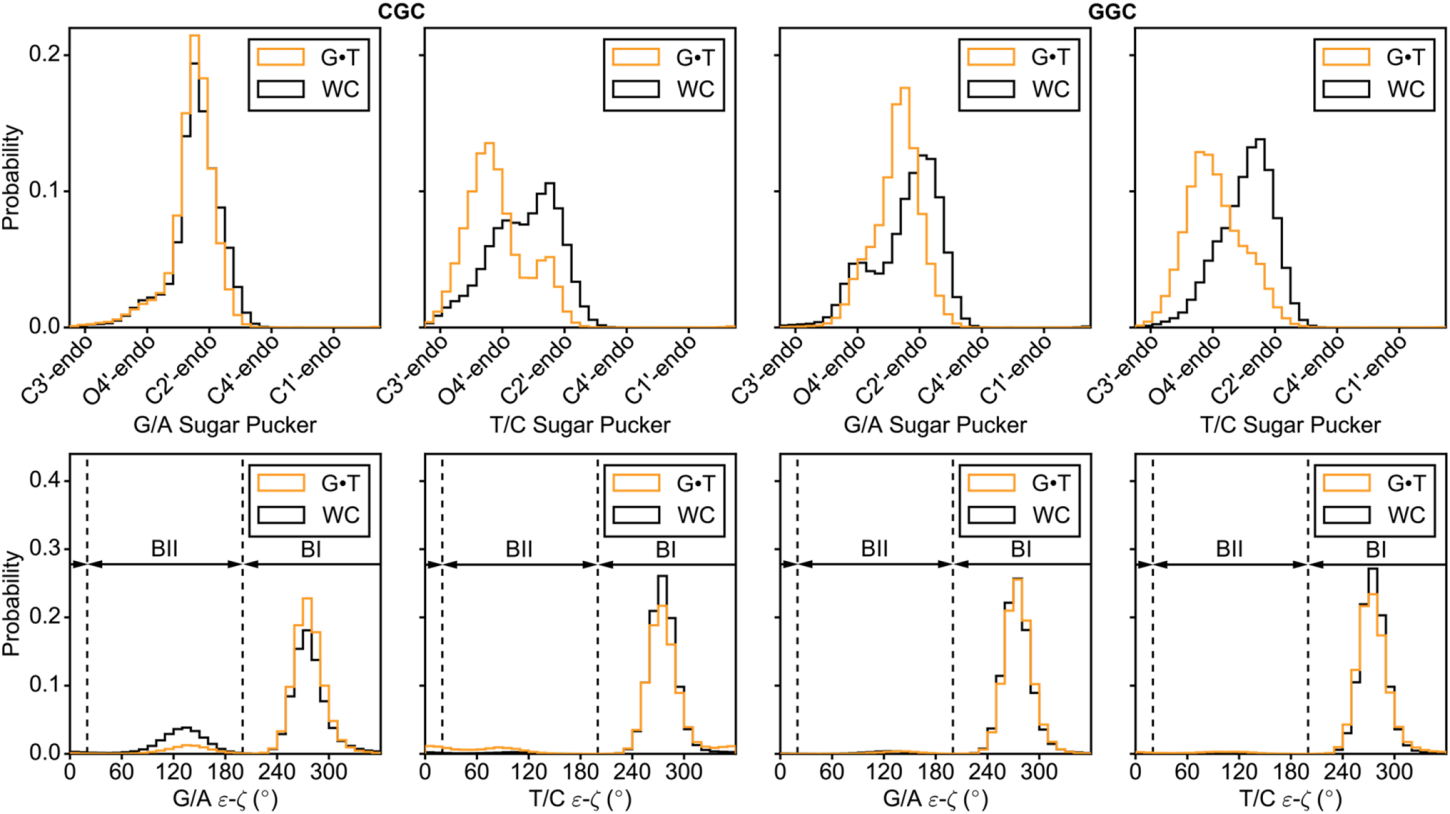
Histograms comparing the sugar pucker and ε−ζ torsion angle for G·T mismatches (orange) and WC bps (black), as obtained from MD simulations on DNA duplexes in two different sequence contexts (CGC and GGC, Supplementary Figs. S2, S3, Materials and Methods). The sugar pucker and torsion angles of the G and T in the G·T mismatch were compared to those of G/A and T/C in WC bps, respectively. The puckers and torsion angles for the WC bps were obtained by averaging over individual simulations with G-C and A-T bps in place of G·T

All crystal structures containing DNA with resolution better than 3 Å as of April 27th 2017 were downloaded from the Protein Data Bank (PDB) (Berman et al. 2000). Structures were analyzed using an in-house Python script to identify wobble G·T mismatches that are flanked by two WC bps on either side to mimic a duplex like environment. A total of 15 G·T mismatches belonging to 14 distinct structures were identified out of a total of 5906 deposited structures in the PDB containing nucleic acids (Supplementary Table S1). The torsion angles and C1′–C1′ distance of the mismatched bps were computed and compared with a set of unmodified G-C and A-T Watson–Crick bps from free (not bound to proteins/ligands) DNA structures placed in a similar structural context with B form helical geometry as determined using DSSR (Lu et al. 2015). The BI/BII phosphate conformation was determined by first converting the ε and ζ torsion angles to the [0°, 360°] range, taking the difference ε−ζ, and then converting the resultant angle into the [0°, 360°] range (see Figs. 2a and 3). A given phosphate was classified as BII if this difference was in the range [20°, 200°], otherwise it was classified as BI.

To define the movements of the G and T bases required to form a wobble G·T mismatch when starting from a WC bp (Fig. 2b), the coordinates for the G·T mismatches along with one WC bp on either side of the mismatch were extracted from the above crystal structures. An idealized triplet of bps with a G-C bp in the middle was constructed using 3DNA (Lu and Olson 2003). Triplets of G·T mismatches were superimposed on the idealized triplet of bps using the sugar atoms of the bps neighboring the mismatch and the idealized G-C bp. An overlay of the G·T mismatches with the idealized G-C bp after superposition is shown in Fig. 2b.

### MD simulations

All MD simulations were performed using the ff99 AMBER force field (Cheatham et al. 1999) with bsc0 corrections for DNA (Perez et al. 2007), using periodic boundary conditions as implemented in the AMBER MD simulation package (Salomon-Ferrer et al. 2013). DNA duplexes with G·T or G-C/A-T WC bps used in the MD simulations (Supplementary Figure S2) were derived from the hairpins (GT-CGC/GT-GGC/AT-CAC/AT-GAC/GC-CGC/GC-GGC) used for the NMR measurements (Supplementary Figure S4) by elongating by two G-C/A-T bps on either end. Starting structures of the WC base paired helices for the simulations were generated by creating idealized B-DNA helices using 3-DNA (Lu and Olson 2003). Corresponding starting structures containing G·T mismatches were obtained by mutating the WC base paired structures. Starting structures were solvated using a truncated octahedral box of SPC/E (Berendsen et al. 1987) water molecules with box size chosen such that the boundary was at least 10 Å away from the DNA atoms. Na^+^ ions treated using the Joung–Cheatham ion parameters (Joung and Cheatham 2008) were then added to neutralize the charge of the system. Minimization, equilibration and production runs (500 ns) were then performed as described previously (Rangadurai et al. 2018). A set of evenly (5 ps) spaced snapshots was used for subsequent analysis using the CPPTRAJ (Roe and Cheatham 2013) suite of programs.

### Sample preparation

#### Unlabeled DNA oligonucleotides

All unlabeled and unmodified DNA oligonucleotides (GT-CGC, GT-GGC, AT-CAC, AT-GAC, GC-CGC and GC-GGC, see Fig. 1c, Supplementary Figure S4) were purchased from Integrated DNA Technologies with standard desalting purification. Unlabeled DNA oligonucleotides containing *N*^1^-methyl deoxy Guanosine (m^1^G, GT-CGC^m1G6^) or deoxyisoGuanosine (isoG, GT-CGC^isoG^ and GT-GGC^isoG^) (Supplementary Figure S4) were synthesized in-house using a MerMade 6 oligo synthesizer. In particular, standard (bz-A, ac-C, n-ibu-G and T) and modified DNA phosphoramidites (n,n-dmf-m^1^G, Chemgenes and dmf-isoG, Glen Research) were used with a coupling time of 1 min, with the final 5′-DMT group being retained during synthesis. The oligonucleotides were then cleaved from the supports (1 μmol, 1000 Å) using ~ 1 mL of AMA (1:1 ratio of ammonium hydroxide and methylamine) for 30 min and deprotected at room temperature for 2 h. The oligonucleotides were then purified using Glen-Pak DNA cartridges followed by ethanol precipitation.

#### Site-labeled DNA oligonucleotides

Site labeled DNA oligonucleotides (slGT-CGC and slGT-GGC) uniformly ^13^C, ^15^N labeled at positions T5 and G15 (Supplementary Figure S4), were purchased from the Yale Keck Oligonucleotide Synthesis Facility with cartridge purification.

#### Fully labeled DNA oligonucleotides

Uniformly ^13^C/^15^N isotopically labeled flGT-CGC (Supplementary Figure S4) was biochemically synthesized using the Zimmer and Crothers method (Zimmer and Crothers 1995). A chemically synthesized DNA hairpin template containing a 3′ ribonucleotide (IDT) was used in addition to Klenow fragment DNA polymerase (New England Biolabs) and ^13^C/^15^N isotopically labeled deoxy nucleoside triphosphates (Silantes). The reaction mixture was centrifuged to remove pyrophosphate and then incubated with NaOH (final concentration 0.3 M) for 3 h at 55 °C for cleavage of the target oligonucleotide from the template. The mixture was then concentrated to 1.5 mL using a 3 kDa molecular weight cutoff centrifugal concentrator (Millipore Sigma). This was followed by addition of 1.5 mL of a formamide based denaturing loading dye. The mixture was then heated at 95 °C for 5 min for denaturation, and then loaded onto a denaturing gel (20% polyacrylamide/8 M urea) for separation of the target oligonucleotide from other nucleic acid species. Gel bands corresponding to the target single strands were identified by UV-shadowing and subjected to electroelution (Whatman, GE Healthcare) followed by ethanol precipitation.

#### Sample annealing and buffer exchange

Oligonucleotides (following ethanol precipitation or as purchased) were re-suspended in water. The samples were annealed by heating at T = 95 °C for ~ 5 min followed by cooling on ice for ~ 1 h. Following annealing, the samples were exchanged three times into the desired buffer using centrifugal concentrators (4 mL, Millipore Sigma) with a 3 kDa molecular weight cutoff. 10% by volume of D_2_O was added to the samples prior to the NMR experiments for establishing C1′-H1′ and C8-H8 assignments (see below). Samples were then lyophilized once and re-suspended in D_2_O for establishing C3′-H3′ and C4′-H4′ assignments (see below) and performing RD measurements unless mentioned otherwise.

#### NMR buffer

Sodium phosphate buffer for NMR measurements was prepared by the addition of equimolar solutions of sodium phosphate monobasic and dibasic salts, sodium chloride, and EDTA to give final concentrations (unless mentioned otherwise) of 15 mM (phosphate), 25 mM and 0.1 mM respectively. The pH was then adjusted by the addition of phosphoric acid or sodium hydroxide, after which the buffers were brought up to the desired volume and filtered and stored for further usage.

### NMR spectroscopy

All NMR experiments were performed on a 700 MHz Bruker Avance 3 spectrometer equipped with a triple-resonance HCN cryogenic probe. All experiments were performed at pH 6.9 and at T = 25 °C in NMR buffer unless stated otherwise. The NMR data was processed and analyzed using NMRpipe (Delaglio et al. 1995) and Goddard and Kneller (2008)

#### Resonance assignments

The sugar C1′-H1′ and base C6-H6/C2-H2/C8-H8 resonances were assigned using 2D [^1^H, ^1^H] Nuclear Overhauser Effect Spectroscopy (NOESY) and 2D [^13^C, ^1^H] Heteronuclear Single Quantum Coherence (HSQC) spectra of samples in 90% H_2_O/10% D_2_O. The C3′-H3′ and C4′-H4′ resonances were assigned using 2D [^1^H, ^1^H] Total Correlation Spectroscopy (TOCSY) and 2D [^13^C, ^1^H] HSQC spectra of samples in 100% D_2_O. Spectra of GT-CGC^m1G6^ were collected at pH 5.0, 25 °C in order to promote formation of the m^1^G6-C14^+^ Hoogsteen bp, while those of GT-CGC^isoG^ and GT-GGC^isoG^ were collected at a lower temperature (pH 6.9, 10 °C) to minimize alternative tautomeric forms of isoG, which are known to be populated at higher temperatures^54^.

#### Off-resonance ^13^C/^15^N R_1ρ_ RD measurements

Off-resonance ^13^C/^15^N *R*_1ρ_ measurements were performed using 1D *R*_1ρ_ schemes employing selective Hartman-Hahn magnetization transfers as described previously (Hansen et al. 2009; Korzhnev et al. 2005; Nikolova et al. 2012). Weak matched ^1^H and ^13^C/^15^N RF fields were used to transfer magnetization selectively from protons to the ^13^C/^15^N nucleus of interest. The longitudinal ^13^C/^15^N magnetization thus produced was allowed to relax for 5 ms to allow equilibration of ground state and excited state populations and then tilted along the appropriate effective field direction. Then a ^13^C/^15^N spin-lock was applied for a maximal duration (< 120 ms for ^15^N and < 60 ms for ^13^C) chosen so as to achieve ∼ 70% loss in signal intensity at the end of the relaxation period. The signal intensity was recorded for 3–10 delays equally spaced over the relaxation period. Spin-lock powers and offsets used are given in Supplementary Table S2. The RD measurements were performed on lyophilized samples in D_2_O at pH 6.9 and T = 25 °C in NMR buffer unless stated otherwise. Use of D_2_O is especially important for C3′/C4′ RD measurements in order to avoid interference from the water signal; for this reason, the C3′ and C4′ RD measurements were performed in in D_2_O unless stated otherwise.

#### Fitting of R_1ρ_ RD data

*R*_1ρ_ values for a given spin-lock power and offset were obtained by extracting the peak intensities as a function of delay time using NMRPipe (Delaglio et al. 1995) and then fitting them to a mono-exponential function (Kimsey et al. 2015). Errors in *R*_1ρ_ (σ_R1ρ_) were estimated using a Monte-Carlo scheme as described previously (Rangadurai et al. 2019). Numerical integration of the Bloch–McConnell (B-M) equations were used to fit *R*_1ρ_ values as a function of spin-lock power and offset to 2-state or 3-state exchange models, by minimizing $\chi^{2} = \sum {\frac{{R_{1,meas} - R_{1,calc} }}{{R_{1} }}}$, using least squares minimization as described previously (Rangadurai et al. 2019). *R*_1ρ,meas_ and *R*_1ρ,calc_ are the measured and calculated *R*_1ρ_ values, respectively, and the summation is over all spin-lock power offset combinations. The fitted exchange parameters for the 2-state individual fits were *p*_B_, *k*_exAB_, *R*_1_, *R*_2_ and Δω, while those for the 3-state individual fits were *p*_B_, *p*_c_, *k*_exAB_, *k*_exAC_, *R*_1_, *R*_2_, Δω_AB_ and Δω_AC_. Global fits were performed by sharing *p*_B_/*p*_c_ and *k*_exAB_/*k*_exAC_ across multiple nuclei.

It has been shown that base carbon and imino nitrogen RD measurements on G·T mismatches can passively sense the WC to Hoogsteen exchange of neighboring bps (Kimsey et al. 2015), a phenomenon that will hereafter be referred to as “Hoogsteen blowback”, in addition to the exchange of the mismatch itself into a WC-like species. In principle, G·T mismatches in GT-CGC and GT-GGC (Fig. 1c, Supplementary Figure S4) can experience Hoogsteen blowback due to both of the neighboring G-C bps, thereby introducing extraneous contributions to the measured RD data. The Hoogsteen blowback contribution from the G6-C14 bp is expected to be significant for T5-C3′ in the G·T mismatch in GT-CGC. This is because formation of Hoogsteen bps is accompanied by sizeable (~ 2 ppm) downfield shifts in the C3′ carbon of the nucleotide 5′ to the purine (Shi et al. 2018). Hoogsteen blowback contributions are expected to be smaller for GT-GGC since none of the mismatched nucleotides are 5′ to the purines of the neighboring WC bps. The WC to Hoogsteen exchange at the G6-C14 bp was quantified by measuring ^13^C *R*_1ρ_ RD on G6-C1′ in flGT-CGC (Supplementary Figs. S4 and S6). The G6-C1′ RD data could be directly fit to the Bloch–McConnell equations assuming a two-state exchange process to characterize the WC to Hoogsteen exchange at the G6-C14 bp. It should be noted that G6-C1′ is not expected to passively sense the transition between wobble and WC-like G·T as ∆ω_G6-C1′_, estimated as the difference in chemical shifts of G6-C1′ between GT-CGC and, GC-CGC or AT-CAC is < ~ 0.3 ppm (Supplementary Figure S5).

Including a shared Hoogsteen blowback exchange process when individually fitting ^13^C/^15^N *R*_1ρ_ RD profiles of the G·T mismatch in slGT-CGC to a 3-state exchange model in a star-like (Trott and Palmer 2004) topology resulted in exchange parameters for the wobble to WC-like exchange that are more similar across different nuclei relative to parameters obtained from 2-state fits which do not account for Hoogsteen blowback (Supplementary Tables S3 and S4, Supplementary Figure S7). Moreover, the ^13^C and ^15^N *R*_1ρ_ RD data for multiple nuclei of the G·T mismatch could also be globally fitted to a 3-state exchange model sharing *p*_*B*_ and *k*_ex_ for the exchange to a WC-like species with the inclusion of a shared Hoogsteen blowback exchange contribution in a star-like (Trott and Palmer 2004) topology (Supplementary Tables S3 and S4, Fig. 5a, Supplementary Figure S9a). In addition, the populations of the WC-like G·T mismatch obtained from RD measurements in 90% H_2_O:10% D_2_O (Supplementary Table S3) and 100% D_2_O (Supplementary Table S4) were in better agreement with each other when globally fitting the data with the inclusion of a shared Hoogsteen blowback exchange process, relative to when blowback was not considered during fitting (Supplementary Figure S8a). Reasonable estimates of ∆ω for the atoms of the G·T mismatch on the formation of a G6-C14 Hoogsteen bp were also obtained from shared fitting of the RD data, that were similar to the difference in chemical shifts of the atoms between GT-CGC^m1G6^ and GT-CGC (Supplementary Tables S3 and S4, Supplementary Figure S8b). The poor agreement seen for T5-C6 is likely due to *N*^1^-methylation of G6 in GT-CGC^m1G6^. RD profiles for slGT-CGC obtained from a global fit with the inclusion of a shared blowback exchange are displayed in Fig. 5a and Supplementary Figs. 6 and 9a. Fits to RD profiles for slGT-GGC to a 2-state exchange model without inclusion of a shared blowback Hoogsteen exchange are shown in Fig. 5b and Supplementary Figure S9b.

Prior studies (Kimsey et al. 2018) have shown that at high pH > ~ 8.0, G·T mismatches can dynamically exchange with tautomeric and anionic states in a star-like or a triangular (Trott and Palmer 2004) exchange topology. The exchange topology for the exchange of the G·T mismatch in slGT-GGC at pH 8.8 and 10 °C was determined by fitting of the ^15^N *R*_1ρ_ data for G15-N1 and T5-N3. Based on the Akaike (Wagenmakers and Farrel 2004) and Bayesian (Burnham and Anderson 2004) information criterion weights, a statistically better fit was obtained for a triangular topology, with populations of the tautomeric and anionic state equal to 0.075 ± 0.005% and 8.019 ± 1.007%, respectively. The pKa ~ 9.9 for the ionization of the G·T mismatch thus obtained was inconsistent with prior estimations (Kimsey et al. 2018) of the pKa ~ 11 made based on ^15^N *R*_1ρ_ data for the G·T mismatch in slGT-GGC at 10 °C at pH values of 8.0 and 8.4, respectively. Thus, the ^15^N and ^13^C *R*_1ρ_ profiles for G15-N1, T5-N3 and T5-C4′ in slGT-GGC at pH 8.8 and 10 °C were fit assuming 3-state exchange between wobble, tautomeric and anionic G·T mismatches in a star-like (Trott and Palmer 2004) exchange topology (Supplementary Figure S11a) instead to yield exchange parameters in Supplementary Table S7. The large error in the fitted Δω_T5-C4′_ for the tautomeric G·T excited state (Supplementary Table S7) is due its low population (0.065 ± 0.003%) and fast exchange rate (4029 ± 352 s^−1^), which results in a small contribution to the observed RD profile, thereby making determination of Δω unreliable.

The uncertainties in the exchange parameters were computed using a Monte-Carlo scheme (Bothe et al. 2014). Alignment of magnetization during B-M fitting was performed as described previously (Rangadurai et al. 2019). Off-resonance *R*_1ρ_ profiles were generated by plotting (*R*_2_ + *R*_ex_) = (*R*_1ρ_−*R*_1_cos^2^ θ)/sin^2^θ, where θ is the angle between the effective field of the observed resonance and the z-axis, as a function of Ω = ω_OBS_−ω_RF_, where ω_OBS_ is the Larmor frequency of the observed resonance and ω_RF_ is the angular frequency of the applied spin-lock.

## Results and discussion

### Wobble G·T mismatches have a distinct sugar-backbone conformation relative to canonical Watson–Crick base pairs

Before examining whether transitions between wobble and WC-like G·T mismatches entail changes in the sugar-backbone conformation, we first examined how and why the sugar-backbone conformation differs for wobble G·T relative to canonical WC G-C or A-T bps. If we were to start with a canonical WC G-C bp, the introduction of a G·T mismatch by replacing the C with a T would result in steric clashes between the imino hydrogen atoms. The steric clashes can be resolved through translation of the two bases by one hydrogen bond register to form a paired wobble G·T mismatch in which the T is displaced toward the major groove relative to the G. It is this movement of the bases to accommodate the wobble pair that will require changes in the sugar-backbone conformation (Allawi et al. 1998; Patel et al. 1982; Hare et al. 1986; Hunter et al. 1987).

To capture the changes in the sugar-backbone conformation required to accommodate the G·T wobble, we surveyed (“Materials and methods” section) existing crystal structures of DNA duplexes containing G·T mismatches. We find that relative to WC G-C and A-T bps, wobble G·T mismatches have sugar puckers that are biased away from C2′-endo towards puckers in between C2′ and C3′-endo, and backbone torsion angles ξ and ζ that are biased away from the BII conformation for both G and T nucleotides (Fig. 2a, Supplementary Figure S1). While it is often assumed that the T (or U) base primarily moves to form a wobble (Westhof et al. 2019; Westhof 2014), the structures show both bases move to form the wobble pair as noted previously for G·T mismatches in A-DNA (Rabinovich et al. 1988) (Fig. 2b).

To further evaluate the robustness of the above results, we carried out 0.5 μs long MD simulations using the parmbsc0 forcefield (Cheatham et al. 1999; Perez et al. 2007) on DNA duplexes containing either G·T, G-C, or A-T bps (see “Materials and methods” section). In agreement with the crystal structure survey, the simulations show changes in the backbone torsion angles ξ and ζ away from the BII conformation at the G of the G·T mismatch, in addition to a bias away from the C2′-endo sugar pucker towards puckers in between C2′ and C3′-endo, at both the G and T of the G·T mismatch (Fig. 3, Supplementary Figure S3).

### Sugar carbon chemical shifts for wobble G·T mismatches differ from canonical Watson–Crick base pairs

The differences in the sugar-backbone conformation, particularly the sugar pucker, between wobble G·T and canonical WC bps (Figs. 2 and 3) are expected to give rise to differences in sugar carbon chemical shifts. The C3′ chemical shift is highly sensitive to sugar pucker while C4′ is sensitive to both sugar pucker and the backbone angle γ (Santos et al. 1989; Fonville et al. 2012; Dejaegere and Case 1998; Xu and Au-Yeung 2000). If these changes in chemical shifts were sizeable (i.e. > 1 ppm), NMR RD measurements targeting sugar C3′/C4′ nuclei of the G·T mismatch could be used to examine whether transitions toward low-abundance short-lived WC-like conformations restore C3′/C4′ chemical shifts and sugar-backbone geometry typical of the canonical WC conformation. It should be noted that based on prior studies (Kimsey et al. 2015) it is not possible to infer the sugar-backbone geometry of WC-like G·T mismatches using the C1′ carbons as probes as they are not sensitive to the wobble to WC-like transition.

**Fig. 4:**
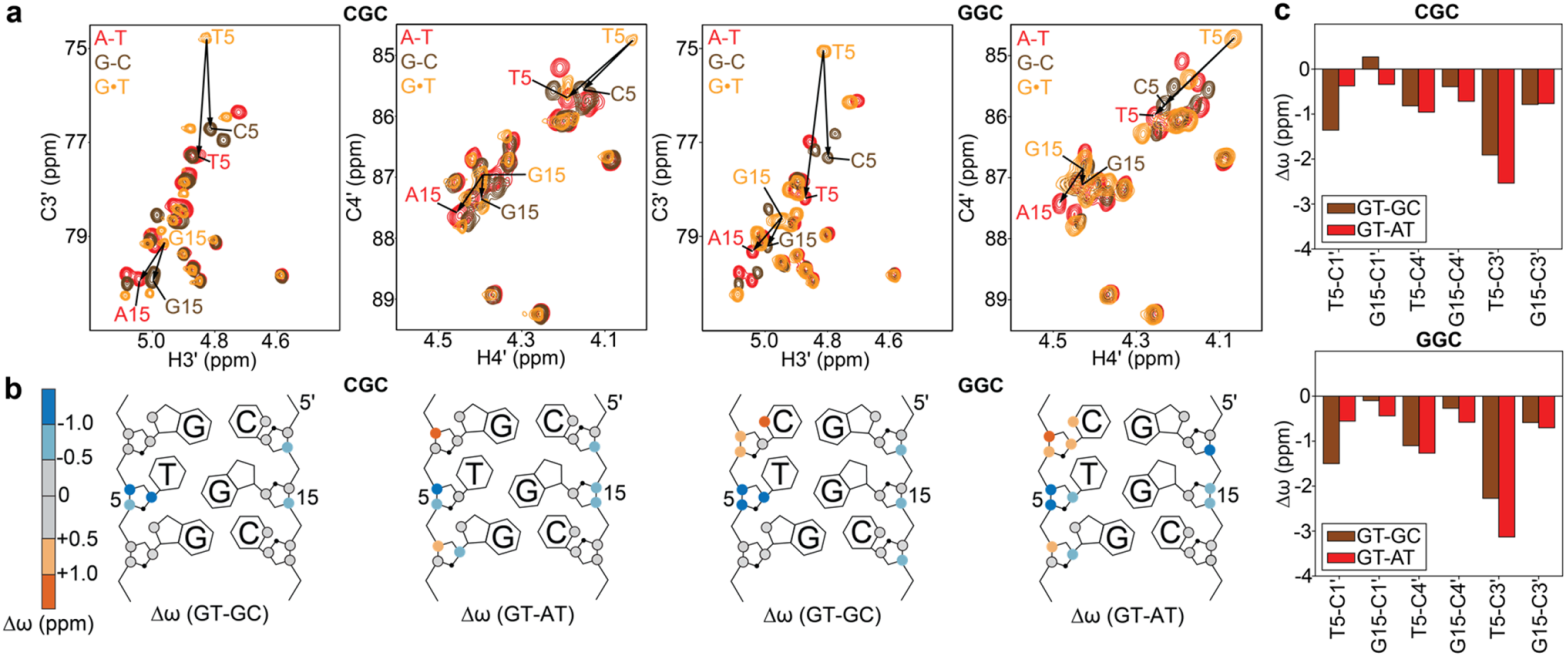
**a** Chemical shift overlays of the C3′-H3′ and C4′-H4′ regions of 2D [^13^C, ^1^H] HSQC spectra for GT-CGC/GT-GGC (yellow), GC-CGC/GC-GGC (brown) and AT-CAC/AT-GAC (red). **b** Chemical shift perturbations induced by the G·T mismatch relative to G-C (GT-GC) or A-T (GT-AT) bps. **c** Bar plot of the Δω for the sugar carbons of the G·T mismatch containing hairpins relative to those containing G-C (brown) or A-T (red) bps

For two different DNA sequence contexts, we compared the sugar C3′ and C4′ chemical shifts of the wobble G·T mismatch with those of G-C and A-T WC bps. We used DNA duplexes containing either G-C or A-T bps in place of G·T (Fig. 1c, Supplementary Figure S4). Indeed, relative to either G-C or A-T bps, wobble G·T mismatches consistently have upfield shifted (~ 1-3 ppm) sugar chemical shifts which are particularly sizeable for G-C3′, T-C3′, and T-C4′ (Fig. 4, Supplementary Figure S5). Such upfield shifted sugar chemical shifts observed for G·T mismatches are exactly as expected for a bias away from a C2′-endo conformation, in agreement with the structure survey (Fig. 2), MD simulations (Fig. 3), prior chemical shift measurements (Shi et al. 2018; Rangadurai et al. 2018; Santos et al. 1989) and Density Functional Theory (DFT) calculations (Fonville et al. 2012; Dejaegere and Case 1998). Additional measurements of ^31^P chemical shifts and *J*_H3′-P_ coupling constants (Gorenstein 1994) are required for characterizing mismatch induced changes in BI/BII phosphate conformations.

Furthermore, the observation of chemical shift perturbations for both the T and G (Fig. 4c) indicates that both residues experience a change in the sugar-backbone on formation of a wobble bp, as expected based on the comparison of crystal structures (Fig. 2b) (Rabinovich et al. 1988). The G·T wobble also induces sizeable chemical shift perturbations in the neighboring WC bps that are particularly large for the 3′ neighbor of the T and the 5′ neighbor of the G (Fig. 4b). These perturbations likely reflect additional potentially destabilizing conformational changes in neighboring bps that help to accommodate the movement of the T and G to form a wobble bp.

### Tautomeric Watson–Crick-like G·T mismatches have sugar chemical shifts that are similar to Watson–Crick base pairs

Based on the above results, transitions between wobble and WC-like G·T mismatches are predicted to lead to large changes (> 1 ppm) in the G-C3′, T-C3′ and T-C4′ chemical shifts if they entail changes in the sugar-backbone to a WC-like conformation. These chemical shift perturbations are large enough to give rise to detectable NMR *R*_1ρ_ RD. In contrast, we do not expect sizeable RD for G-C4′ because the changes in chemical shifts are predicted to fall near or below the RD detection limit (< 1 ppm).

**Fig. 5:**
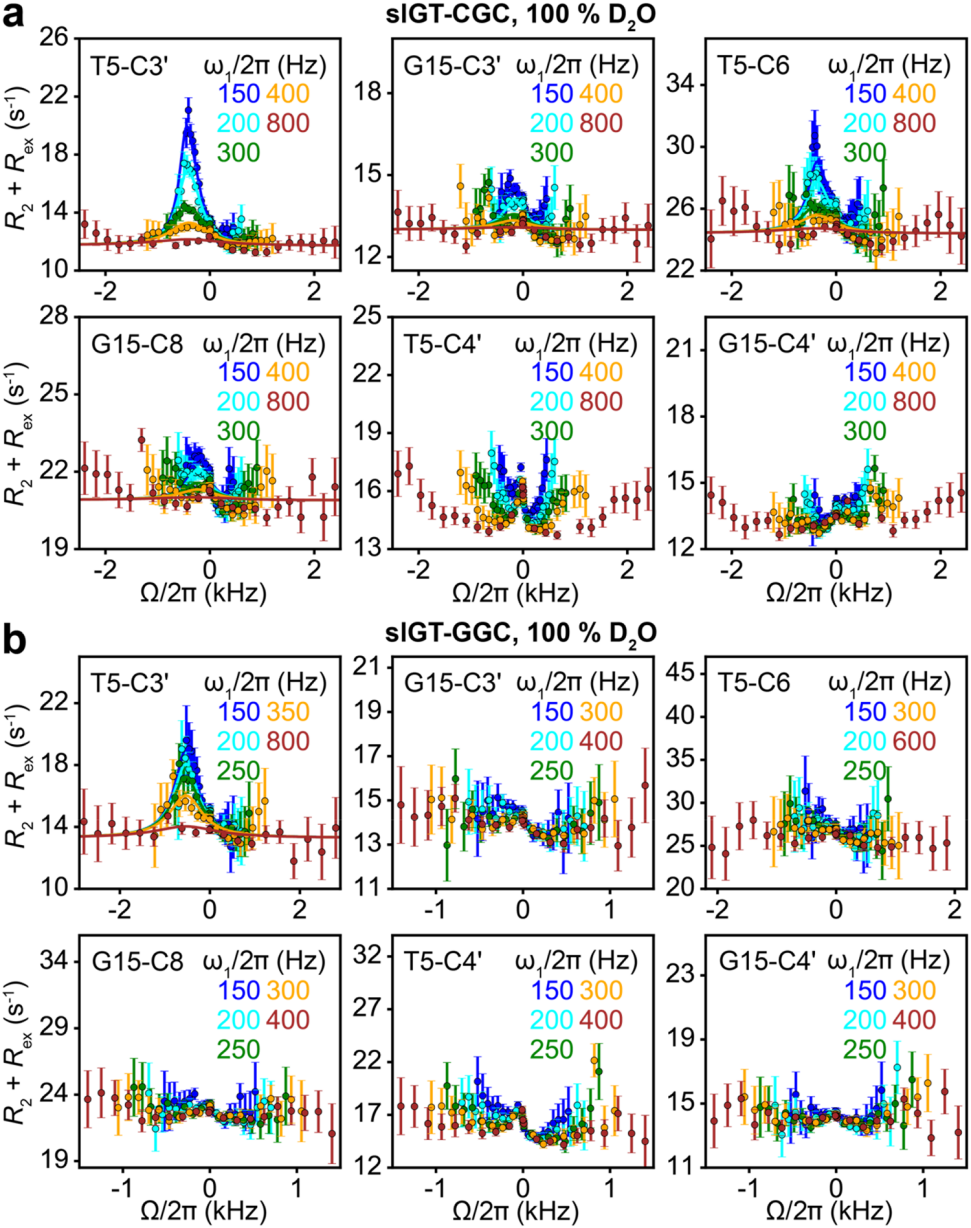
^13^C off-resonance *R*_1ρ_ RD profiles measured for **a** slGT-CGC and **b** slGT-GGC hairpins at pH 6.9 and 25 °C in 100% D_2_O. Shown are experimental data points with a global fit to the Bloch–McConnell equations (solid lines) assuming a 3-state exchange process in a star-like (Trott and Palmer 2004) topology with shared exchange parameters for the wobble to WC-like exchange of the G·T mismatch and the Hoogsteen blowback exchange contribution for slGT-CGC, and a fit to a 2-state exchange model for slGT-GGC (Materials and Methods). Error bars represent the experimental uncertainty of the *R*_1ρ_ data and were computed as described previously by propagating the experimental error in *R*_1ρ_ (Rangadurai et al. 2019). Spin-lock powers are color-coded

We tested the above predictions by measuring off-resonance *R*_1ρ_ RD profiles for G15-C3′, G15-C4′, T5-C3′ and T5-C4′ in the slGT-CGC and slGT-GGC DNA hairpins (Fig. 5, Supplementary Tables S4 and S6, “Materials and methods” section) at pH 6.9 and T = 25 °C in D_2_O. Under these pH conditions, any contribution to the RD profiles from the transient formation of the anionic G·T¯ mismatch (*p*_B_ ~ 0.001%) can be safely ignored. Thus, RD profiles are expected to sense the two state exchange between wobble and tautomeric WC-like G·T mismatches. Experiments were performed in D_2_O to minimize interference from the water signal (“Materials and methods” section). To allow comparison with prior results (Szymanski et al. 2017; Kimsey et al. 2018; Kimsey et al. 2015), we also measured ^15^N and ^13^C *R*_1ρ_ RD profiles for G-N1, T-N3, G-C8 and T-C6 in water (Supplementary Figure S9, Supplementary Tables S3 and S5), as well as for G-C8 and T-C6 carbons, which are known probes for the wobble to WC-like transition, in D_2_O (Fig. 5, Supplementary Table S4).

As predicted, sizeable off-resonance *R*_1ρ_ RD profiles were observed for G15-C3′ and T5-C3′ in slGT-CGC, and T5-C3′ in slGT-GGC whereas the profiles were weaker and in most cases undetectable for T5-C4′ and G15-C4′ (Fig. 5). The lack of observed RD for G15-C3′ in slGT-GGC is likely due to its small Δω (~ 1 ppm) coupled with its faster rate of exchange (1657 ± 189 s^−1^) to form a WC-like species relative to slGT-CGC (909 ± 142 s^−1^, Supplementary Tables S4 and S6). The lack of observable RD for T5-C4′ in both slGT-CGC and slGT-GGC is likely because of the small Δω (~ 1 ppm, see Fig. 4c).

The C3′ RD profiles could be fit satisfactorily to exchange models involving a dominant ground state (GS) and a sparsely populated excited state (ES) (Mulder et al. 2001) (Fig. 5, Supplementary Figs. S6–S8, “Materials and methods” section). The fits yielded exchange parameters of interest, including the ES population (*p*_ES_), the GS↔ES exchange rate (*k*_ex_ = *k*_1_ + *k*_-1_, where *k*_1_ and *k*_-1_ are the forward and backward rate constants), and the chemical shift difference between the ES and GS (Δω = ω_ES_−ω_GS_), which carries structural information.

**Fig. 6:**
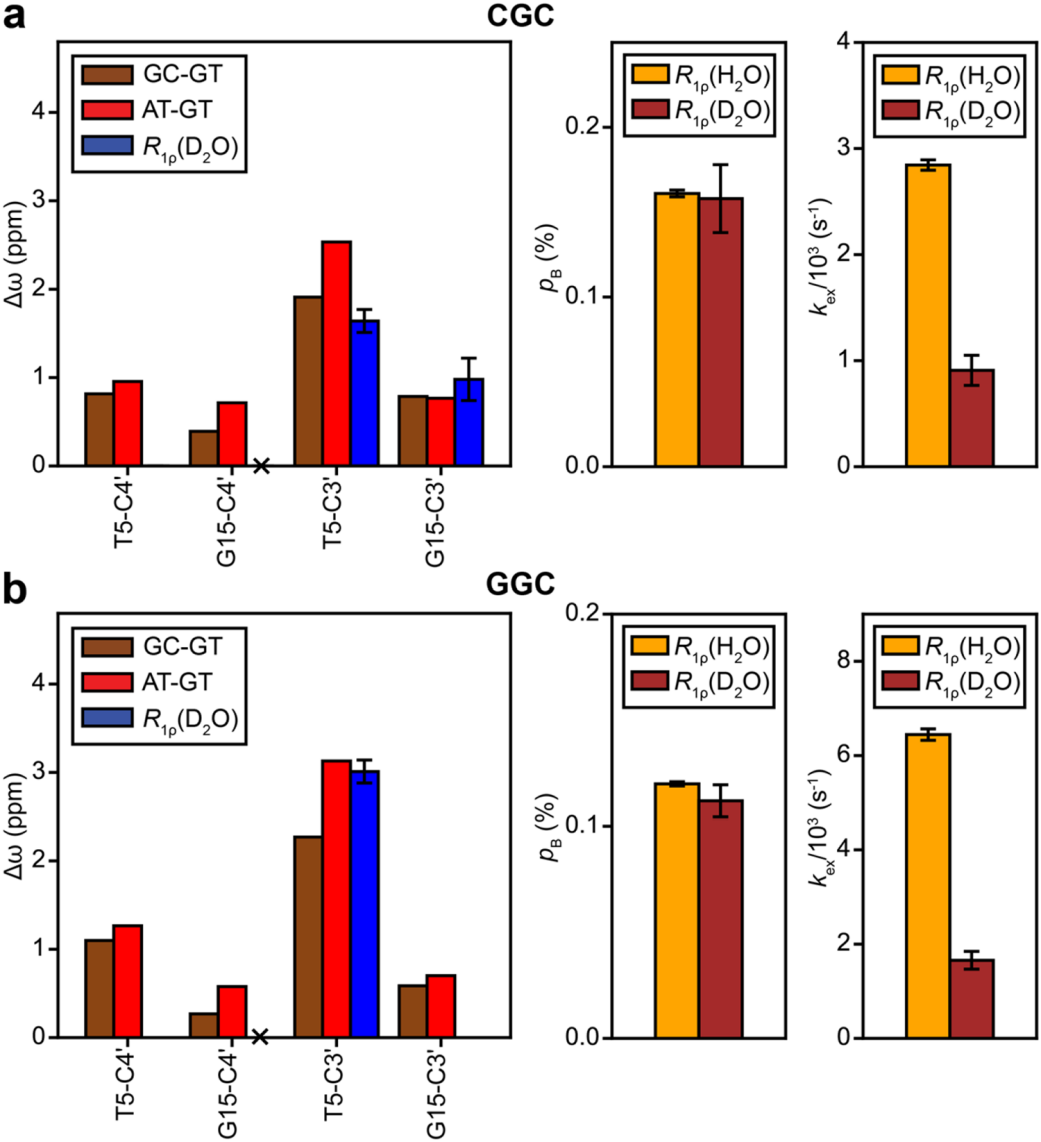
Comparison of Δω of the sugar carbons for the formation of a WC-like G·T mismatch, estimated as the difference in chemical shifts between hairpins containing G-C and G·T bps (brown), A-T and G·T bps (red) and those obtained from ^13^C *R*_1ρ_ measurements in 100% D_2_O (blue). Nuclei for which Δω < 0.5 ppm are not expected to show detectable RD and are indicated using a ‘X’ symbol. Also shown are *p*_B_ and *k*_ex_ for the formation of WC-like G·T mismatches obtained using ^13^C and ^15^N *R*_1ρ_ measurements in 90% H_2_O:10% D_2_O (yellow) and ^13^C *R*_1ρ_ measurements in 100% D_2_O (brown). The data is shown for sequence contexts CGC (**a**) and GGC (**b**). Error bars denote uncertainty in the fitted parameters determined using a Monte-Carlo scheme (Bothe et al. 2014) when fitting the *R*_1ρ_ data for slGT-CGC to a 3-state exchange model with shared exchange parameters, and the data for slGT-GGC to a 2-state exchange model, as described in the Materials and Methods

The Δω values deduced from fitting the sugar C3′ RD data were in excellent agreement with those expected for a transition between the wobble and WC-like conformation, estimated as the difference between the chemical shifts of WC G-C/A-T bps and wobble G·T mismatches (Fig. 6). In addition, the ES populations were in very good agreement with values obtained independently from fitting base G-C8 and T-C6, and imino nitrogen G-N1 and T-N3 RD data in H_2_O (Kimsey et al. 2018; Kimsey et al. 2015) (Fig. 6, Supplementary Figure S8a, Supplementary Tables S3–S6). However, the *k*_ex_ measured in D_2_O was ~ 3.5 fold slower than values measured in H_2_O.

Based on DFT studies (Nomura et al. 2013; Brovarets^2015^), the transition state for conversion between wobble and tautomeric WC-like G·T entails movement of a proton from G-O6 to T-O4 (Supplementary Figure S10). If this were the case, we would expect a ~ 2-4 fold reduction in the rate of the reaction in D_2_O versus H_2_O (Anslyn 2005). Such isotope effects have previously been reported in studies of conformational changes involving proton transfer in proteins (Watt et al. 2007). The reduced *k*_ex_ in D_2_O versus H_2_O therefore provides the first experimental evidence in support of a transition state for tautomerization involving proton transfer. While additional experiments including measurement of RD as a function of viscosity are needed to rule out that the observed change in *k*_ex_ is not due to a change in viscosity between D_2_O and H_2_O (Watt et al. 2007), this highlights the general utility of kinetic isotope effects in the RD-based characterization of nucleic acid transition states. Taken together, the sugar RD data shows that the sugar-backbone adopts a WC-like conformation when forming the tautomeric WC-like G·T mismatch.

### Probing the sugar conformation of anionic G·T^¯^ base pair

**Fig. 7:**
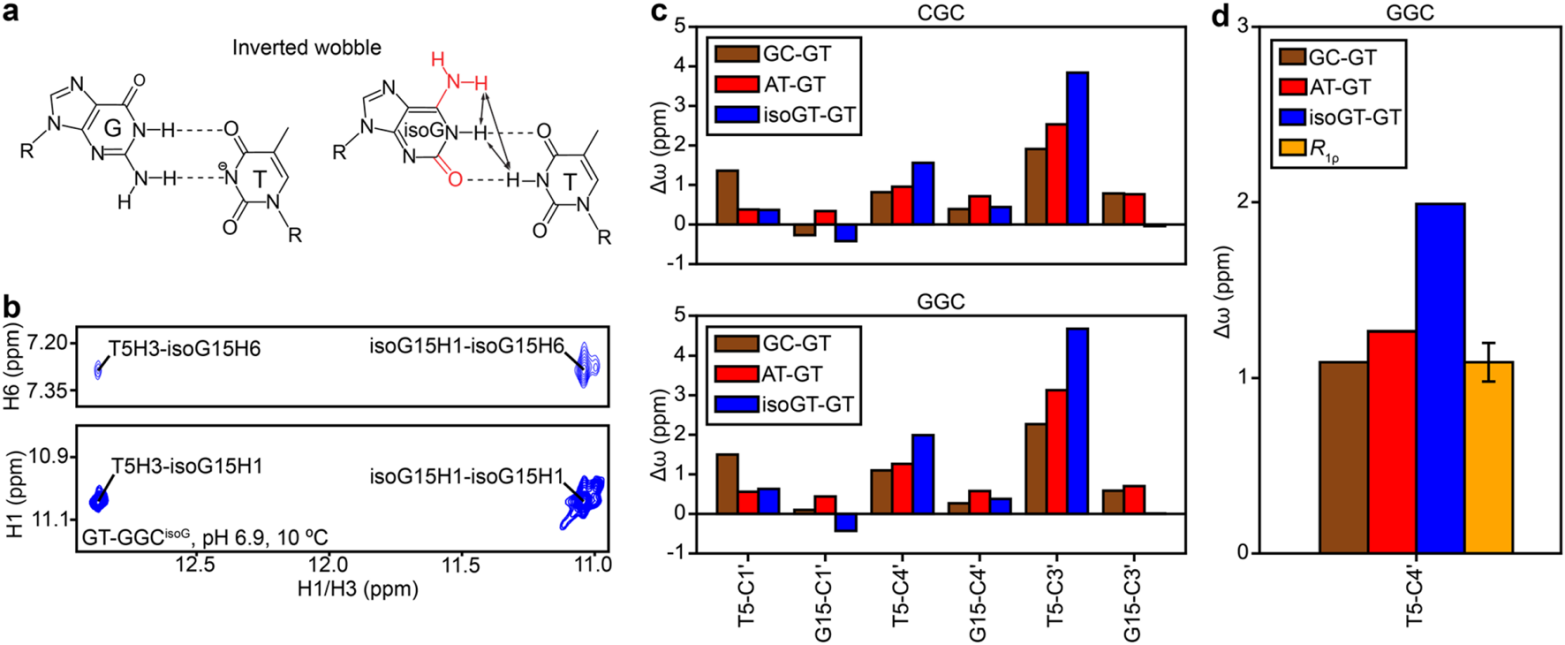
**a** Chemical structures of G·T^¯^ and isoG·T inverted wobble bps. Chemically modified sites in isoG are indicated in red. Arrows on the isoG·T bp denote characteristic NOE connectivities expected for the inverted wobble. **b** 2D [^1^H,^1^H] NOESY spectrum of the imino–imino and imino-amino region for GT-GGC^isoG^. **c** Comparison of Δω of the sugar carbons for the formation of a WC-like G·T mismatch, estimated as the difference in chemical shift between hairpins containing G-C and G·T bps (brown) or A-T and G·T bps (red). Also shown is the Δω for the formation of an inverted wobble G·T^¯^ mismatch, estimated as the difference in chemical shift between hairpins containing isoG·T and G·T bps (blue). **d** Comparison of Δω for T5-C4′ for the formation of a WC-like (brown and red) or inverted wobble G·T mismatch (blue), with the experimental value for the G·T^¯^ ES obtained from *R*_1ρ_ measurements (yellow) on GT-GGC at pH 8.8 and 10 °C in 90% H_2_O:10% D_2_O (Materials and Methods). The uncertainty in Δω denotes the fitting error estimated using a Monte-Carlo scheme as described previously (Bothe et al. 2014)

We also used sugar RD to probe the sugar-backbone conformation of the anionic G·T^¯^ ES observed at high pH (Kimsey et al. 2018; Kimsey et al. 2015). Ionization of T at high pH (> 8.0) was previously inferred based on the appearance of a second pH dependent ES with a large (~ 50 ppm) downfield shift in T-N3 (Kimsey et al. 2018; Kimsey et al. 2015). However, in addition to a WC-like conformation, a G·T^¯^ mismatch can also form an ‘inverted wobble’ conformation in which the bases move by one additional hydrogen bond register past WC pairing (Fig. 7a). Inverted G·T wobble bps have previously been observed crystallographically (Johnson and Beese 2004; Xia and Konigsberg 2014) and more recently, G·U inverted wobbles have been observed in the context of modified bases at the wobble position of the tRNA-mRNA mini-helix in the ribosome (Rozov et al. 2016). The inverted wobble was not suggested to contribute significantly to the anionic G·T^¯^ ES based on DFT calculations in a prior study (Kimsey et al. 2015) .The measured Δω (56 ppm) of T-N3 for the anionic ES was in good agreement with DFT calculations assuming a Watson–Crick like ES (Δω = 54 ppm), and was different from the calculated Δω for an inverted wobble (Δω = 46 ppm). However, direct experimental data is needed to rule out the possibility that the ES observed at high pH is a WC-like conformation not an inverted wobble.

Given the greater movement of the bases in forming an inverted wobble relative to WC, we hypothesized that the inverted wobble has a unique sugar-backbone conformation, and in turn, unique sugar chemical shifts distinct from those of canonical WC bps. If this were the case, sugar RD data could be used to rule out an inverted anionic wobble G·T^¯^ ES. We used G to isoguanosine (isoG) substitution (Robinson et al. 1998) to stabilize an inverted wobble conformation (Fig. 7a) and thereby deduce its sugar chemical shifts. While the chemical modification of the G and lack of ionization of the T would not be expected to faithfully reproduce the base chemical shifts of the inverted wobble G·T^¯^, the sugar chemical shifts should not be significantly affected by chemical modification of the base (Xu and Au-Yeung 2000).

2D [^1^H, ^1^H] NOESY spectra showed cross peaks between T-H3, isoG-H1 and isoG-H6 as expected for an inverted wobble isoG·T bp (Fig. 7b). Furthermore, 2D HSQC spectra (Supplementary Figure S5) revealed that the T-C3′ and the T-C4′ sugar carbon chemical shifts are more downfield shifted for the inverted wobble relative to the WC conformation. Therefore, transition of a wobble G·T to an inverted wobble G·T^¯^ is expected to result in a larger downfield chemical shift for T-C3′ and T-C4′ relative to a transition toward the WC-like G·T^¯^ conformation (Fig. 7c). In contrast, the other sugar carbon chemical shifts (T-C1′, G-C1′, G-C3′, G-C4′) were small (< 1 ppm) and/or similar to the shifts expected for a WC bp (Fig. 7c), and therefore were less suited for distinguishing inverted wobble versus WC-like G·T^¯^ conformations using RD.

We therefore chose T-C3′ and T-C4′ as RD probes as they should in theory be able to distinguish between the inverted wobble and WC-like G·T^¯^ conformation. In principle, one can obtain these sugar chemical shifts for G·T^¯^ by fitting the sugar RD data measured at high pH. However, in practice, owing to the small magnitude of the exchange contributions in the RD profiles, fitting the sugar RD data to a 3-state exchange process involving the wobble, tautomeric and anionic species yields unreliable estimates for the exchange parameters, including the chemical shifts. The exchange parameters can be more reliably determined with the inclusion of RD data from G-N1 and T-N3 RD owing to their much larger exchange contributions in the RD profiles, which is a consequence of the large changes in chemical shifts during tautomerization and ionization (Kimsey et al. 2018). This in turn necessitates that the RD be measured in H_2_O. While this complicates C3′ RD measurements owing to the fact that the H3′ protons are close to the water resonance frequency, this is not an issue for the C4′ RD measurements as the H4′ protons are relatively farther away from water. Thus, our strategy to rule out the inverted wobble conformation for the G·T^¯^ anionic ES involved performing imino nitrogen and T-C4′ RD measurements in H_2_O.

The imino G15-N1 and T5-N3, and sugar T5-C4′ RD profiles for slGT-GGC could be globally fitted together to a 3-state exchange process between wobble, tautomeric and anionic ES G·T bps in a star-like topology (Kimsey et al. 2018; Trott and Palmer 2004) (Supplementary Figure S11a, “Materials and methods” section) to obtain exchange parameters (Supplementary Table S7). The difference in chemical shifts between the GS and anionic ES for T-C4′ was found to be in better agreement with that expected for formation of a WC-like bp as compared to an inverted wobble (Fig. 7d). Moreover, the quality of the fit worsened when fixing the Δω to be equal to the value expected for an inverted wobble G·T^¯^ bp (Supplementary Figure S11b). These data indicate that the anionic G·T^¯^ ES has a WC-like conformation and help to rule out the alternative inverted wobble conformation.

## Discussion

Determining the structures of short-lived low-abundance excited conformational states of biomolecules, such as the WC-like G·T mismatches studied in this work presents a significant challenge to conventional biophysical methods. NMR RD experiments provide a rare opportunity to detect such ESs as well as to probe their conformations (Rangadurai et al. 2019; Sekhar and Kay 2013; Korzhnev and Kay 2008; Hansen et al. 2008; Xue et al. 2015; Zhao and Zhang 2015). In both proteins and nucleic acids, measurements of RD data for a variety of spins is crucial for accurately deducing the ES conformation (Shi et al. 2018; Sathyamoorthy et al. 2017; Hansen et al. 2008). Prior studies (Shi et al. 2018; Clay et al. 2017) have highlighted the utility of sugar RD data in characterizing ES Hoogsteen bps in DNA and ESs involving alternative secondary structures in RNA. In this study, the sugar RD data could be used to obtain new evidence in support of an ES that involves tautomeric and anionic WC-like G·T conformations. It should be noted that the lack of detectable RD on the C1′ carbons of the G·T mismatch, in spite of the known sensitivity of the C1′ chemical shift to changes in sugar pucker (Santos et al. 1989; Fonville et al. 2012; Dejaegere and Case 1998; Xu and Au-Yeung 2000) that accompany formation of a WC-like mismatch (Figs. 2 and 3) is likely due to compensatory changes in χ angle (Supplementary Figs. 1 and 3). Such compensation effects the χ-angle and sugar pucker have previously been noted in the context of RNA (Zhou et al. 2016) and DNA (Shi et al. 2018).

While it is not surprising that tautomeric WC-like G·T mismatches adopt a WC-like sugar-backbone conformation, the results help to rule out an inverted wobble conformation for the case of anionic G·T^¯^ mismatches. The WC-like sugar-backbone conformation further supports a mutagenic role for these fleeting WC-like states as this is likely to align functional groups for phosphodiester bond formation during catalysis by DNA polymerases. Based on these results, it is very likely that base modifications that stabilize ionization and promote miscoding such as 5-Bromo and Fluoro uridine (Yu et al. 1993), and Uridine 5-oxyacetic acid (Strebitzer et al. 2018) do so in part via formation of a WC-like ES conformation.

Our studies add to a growing view that in duplex DNA, non-canonical bps such as Hoogsteen and wobble G·T mismatches have sugar puckers that are biased away from the canonical C2′-endo conformation. In this regard, it is interesting to note that prior NMR studies indicated that the sugar pucker at wobble G·T mismatches is C2′-endo (Allawi and SantaLucia 1998; Hare et al. 1986) and not biased away from the C2′-endo conformation as inferred here based on analysis of chemical shifts and MD simulations. This discrepancy is likely due to the absence of *J*-coupling restraints for constraining the sugar pucker during structure refinement in the study by Hare et al. (Hare et al. 1986) and possibly due to differences in sequence context in the study by Allawi and SantaLucia (1998). It is also interesting to note that changes in backbone angles and movements of the bases required for the formation of wobble G·T mismatches identified in this study (Figs. 2 and 3) qualitatively mirror those recently described for non-canonical A-T Hoogsteen bps (Shi et al. 2018; Sathyamoorthy et al. 2017) in which the bases have to come into proximity without changes in the hydrogen bond register

Crystal structures of some DNA polymerases in complex with DNA, especially those belonging to the A-family, show that the WC base paired DNA near the incoming correct base pair bp typically adopts a conformation intermediate between A and B forms, with C3′-endo sugar puckers and a wide minor groove (Kiefer et al. 1998; Eom et al. 1996; Batra et al. 2006). Thus, further studies of G·T/G·U mismatches in analogous environments such as RNA–DNA hybrids or A-RNA could shed light into the nature of the sugar-backbone changes required for formation of WC-like G·T mismatches in these contexts, and consequently DNA conformational changes required for misincorporation by these DNA polymerases. Additional studies are also needed to characterize DNA sugar-backbone dynamics within the physiologically relevant polymerase active site environment. The sugar RD data can also be measured in flexible environments mimicking physiological substrates of DNA polymerases such as mismatches at the terminal ends, where ^15^N base RD measurements may be compromised due to exchange of the imino protons with solvent. More generally, the approach outlined in this study can also be extended to other mismatches to probe their mutagenic conformations, especially given the difficulties with observing imino signals in mismatches such as A·C (Gao and Patel 1987; Patel et al. 1984).

## Electronic supplementary material

Below is the link to the electronic supplementary material.

Supplementary material 1 (DOCX 9672 kb)
