# Supplementary material for: Probing conformational transitions towards mutagenic Watson–Crick-like G·T mismatches using off-resonance sugar carbon R1ρ relaxation dispersion
Source: J Biomol NMR. 2020 Aug 12;74(8):457–71. doi: 10.1007/s10858-020-00337-7 (PMC7508749; doi:10.1007/s10858-020-00337-7)
Supplement: Supplementary file 1 — Supplementary material 1 (DOCX 9672 kb) [file 10858_2020_337_MOESM1_ESM.docx]

**Supplementary Information**

**Probing conformational transitions towards mutagenic Watson-Crick-like G•T mismatches using off-resonance sugar carbon *R*_1ρ_ relaxation dispersion**

Atul Rangadurai^1^, Eric Szymanski^1,2^, Isaac Kimsey^1,2^, Honglue Shi^3^, and Hashim M. Al-Hashimi^1,3^*

^1^ Department of Biochemistry, Duke University School of Medicine, Durham, NC, 27710, USA

^2^ Present address Nymirum, 4324 S. Alston Avenue, Durham, NC, 27713, USA

^3^ Department of Chemistry, Duke University, Durham, NC, 27710, USA


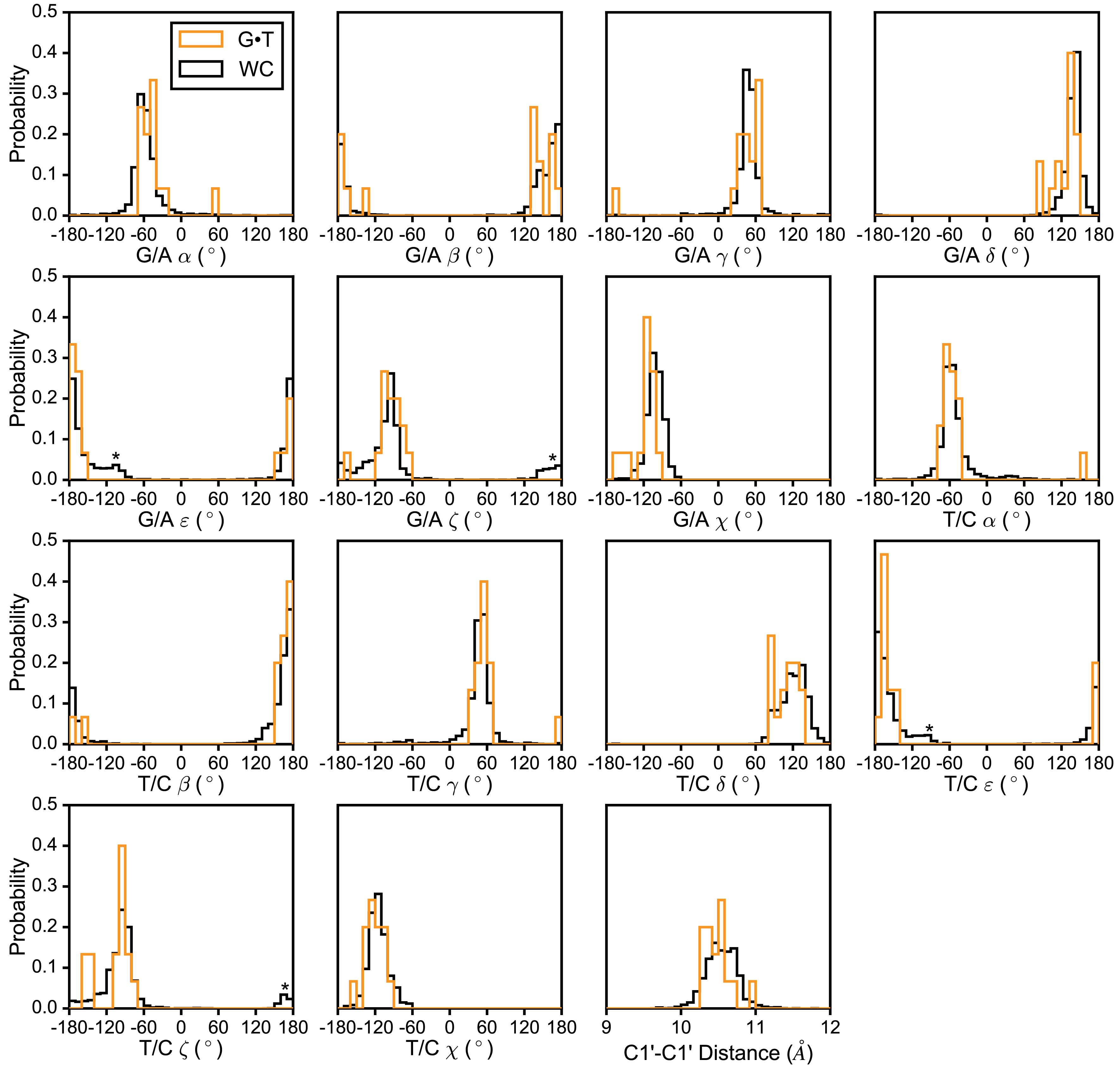


**Supplementary Figure 1** Histogram of the endocyclic torsion angles and the C1'-C1' distance for wobble G•T mismatches (orange) and G-C and A-T WC bps (black), as obtained froma survey of crystal structures in the PDB. The torsion angles of the G and T in the wobble G•T mismatch were compared to those of G/A and T/C in WC bps, respectively. * denotes values of ε and ζ torsions that correspond to the adoption of a BII phosphate conformation.


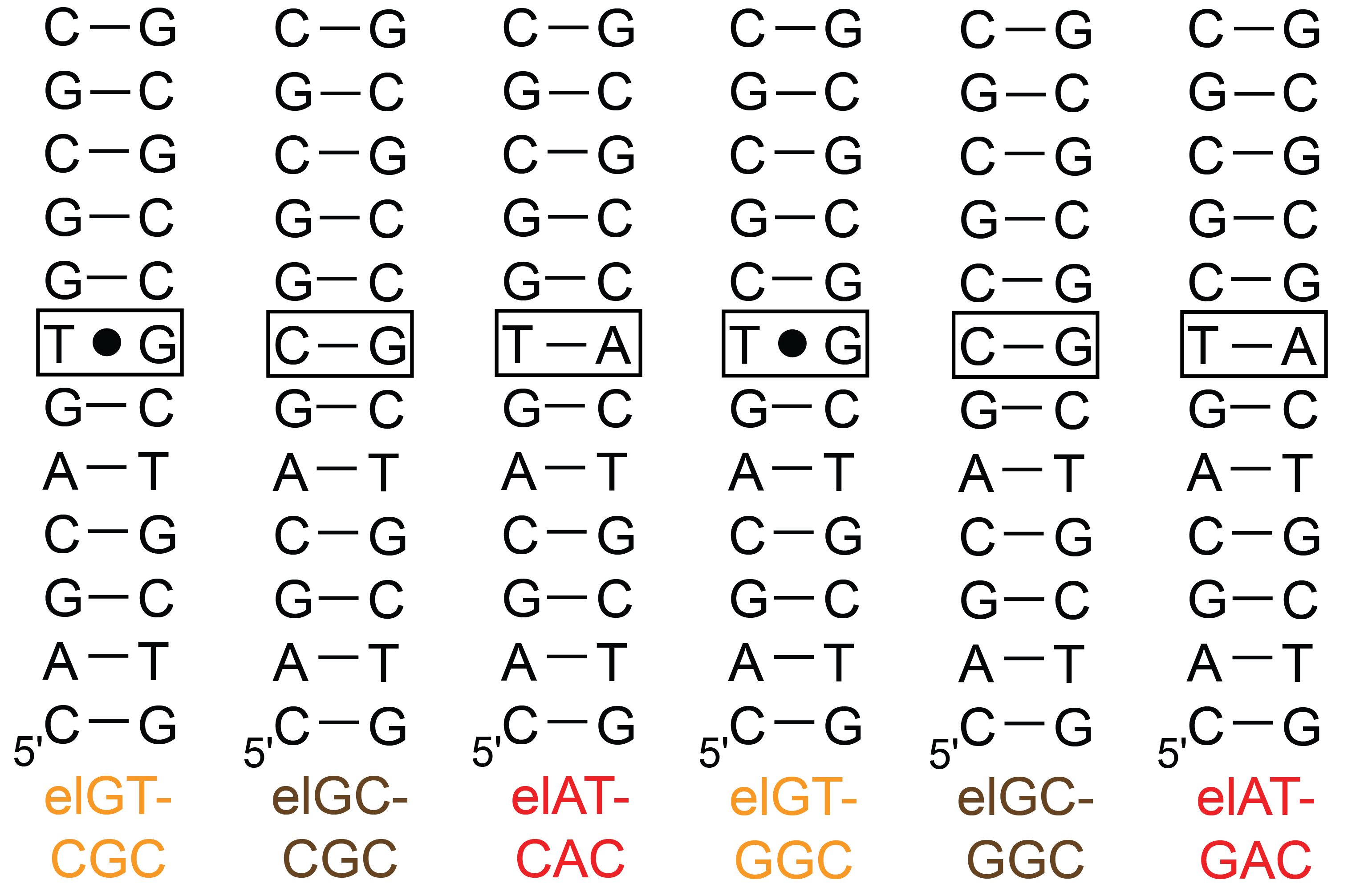


**Supplementary Figure 2** DNA constructs used for MD simulations. el denotes elongated


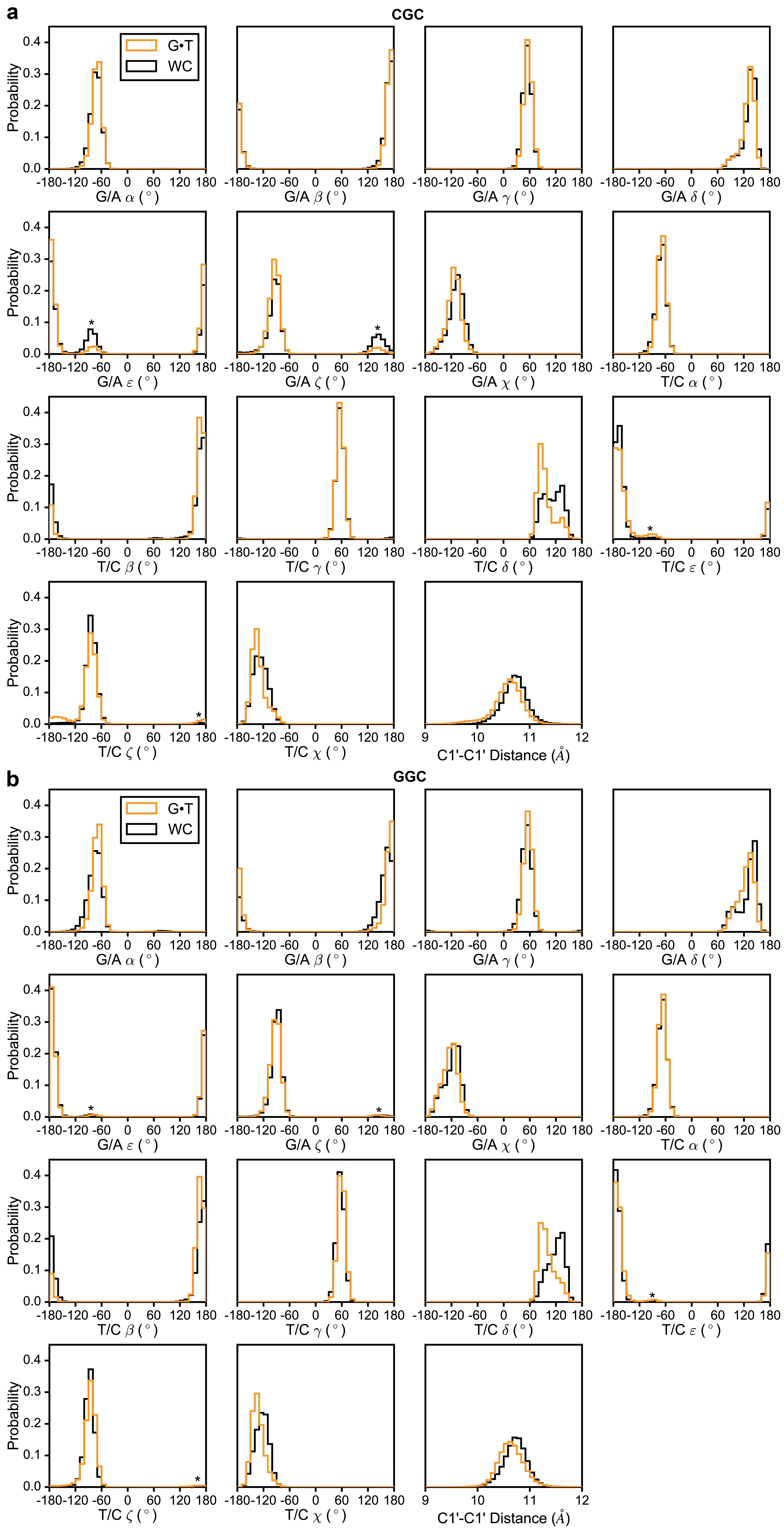


**Supplementary Figure 3** Histogram of the endocyclic torsion angles and the C1'-C1' distance for wobble G•T mismatches (orange) and G-C and A-T WC bps (black), as obtained from MD simulations on DNA duplexes in two different sequence contexts CGC (a) and GGC (b) (Supplementary Figure 2, Materials and Methods). The torsion angles of the G and T in the G•T mismatch were compared to those of G/A and T/C in G-C and A-T WC bps, respectively. The torsion angles and C1'-C1' distance for the WC bps were obtained by averaging over individual simulations of duplexes with G-C and A-T bps in place of G•T (Supplementary Figure 2). * denotes values of ε and ζ torsions that correspond to the adoption of a BII phosphate conformation.


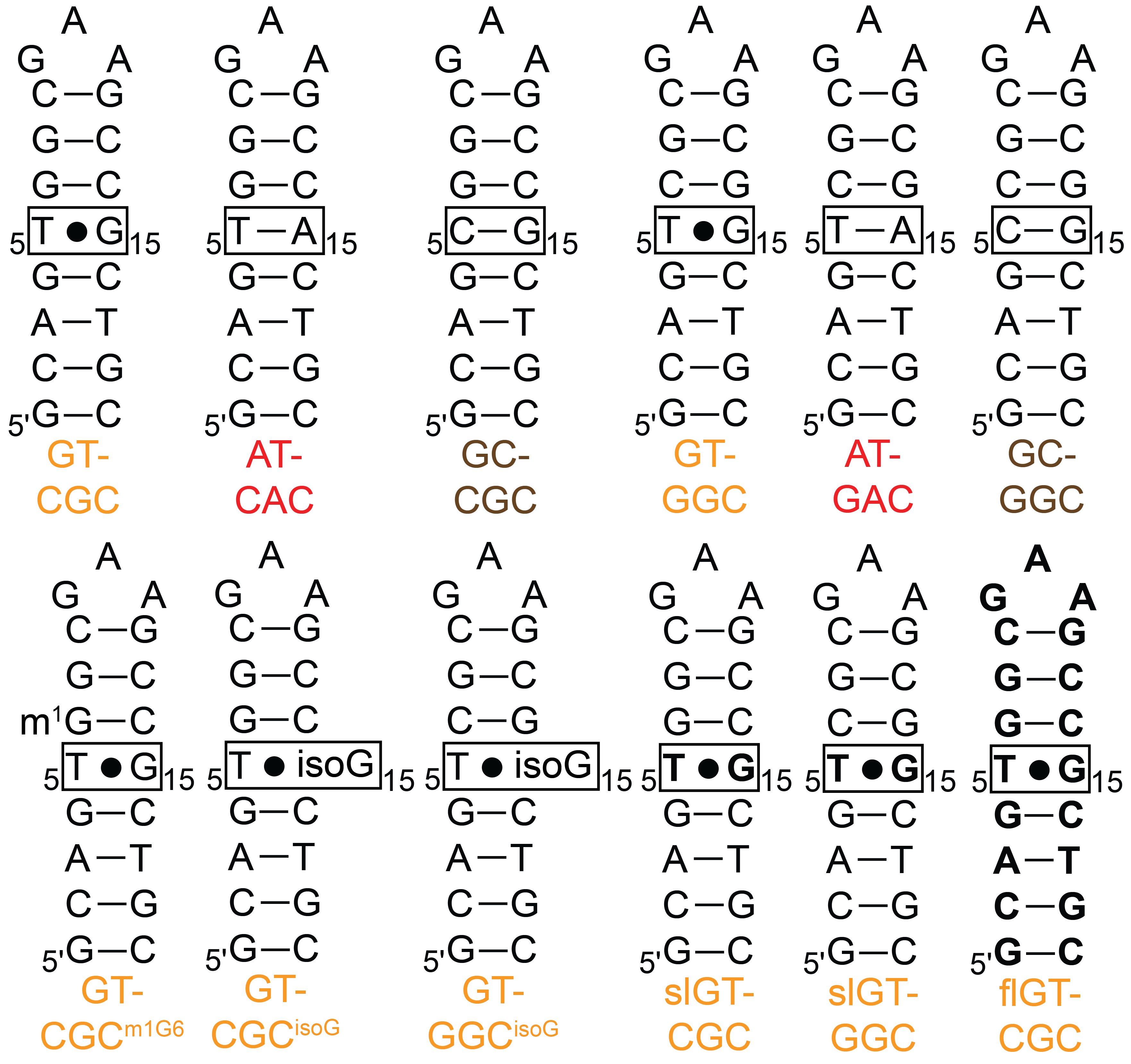


**Supplementary Figure 4** DNA constructs used in this study. Nucleotides in bold are uniformly ^13^C, ^15^N isotopically labeled while all other nucleotides are unlabeled. m^1^G denotes *N*^1^-methyl deoxyguanosine, isoG denotes deoxyisoGuanosine. sl denotes site labeled and fl denotes fully labeled

**

**

**

**

**
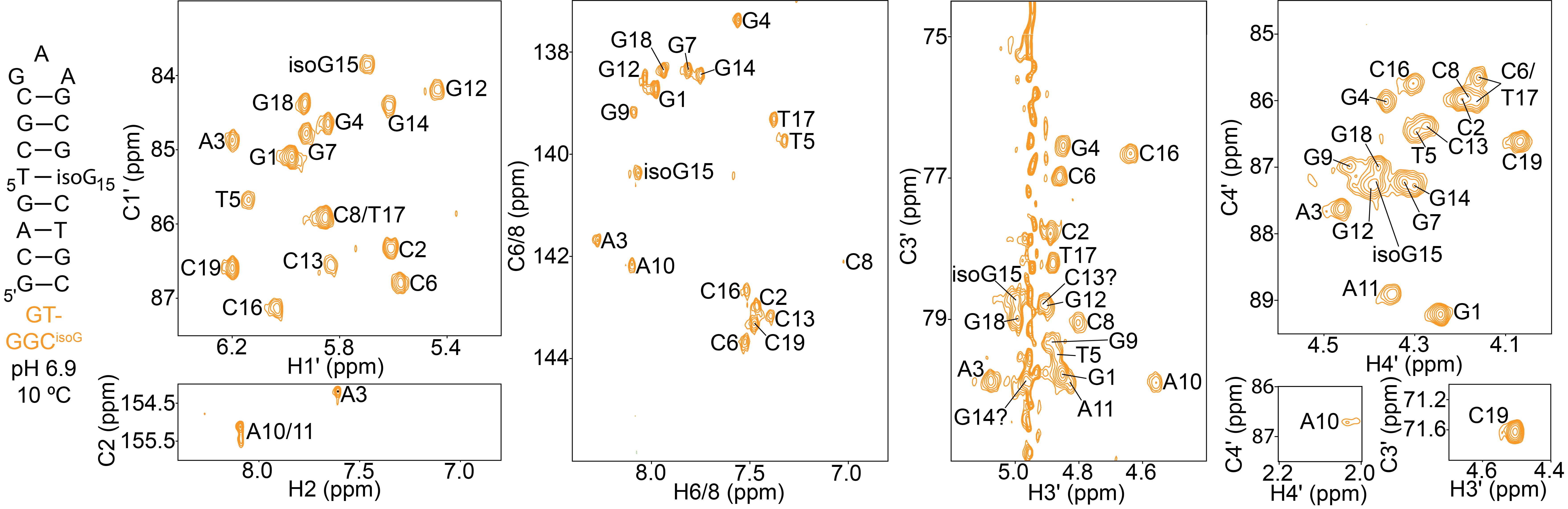
**

**Supplementary Figure 5** 2D [^13^C, ^1^H] HSQC spectra showing the resonance assignments for C1'-H1', C2-H2, C6-H6/C8-H8, C3'-H3' and C4'-H4' in the various DNA constructs used in this study. All spectra were collected in 100 % D_2_O.


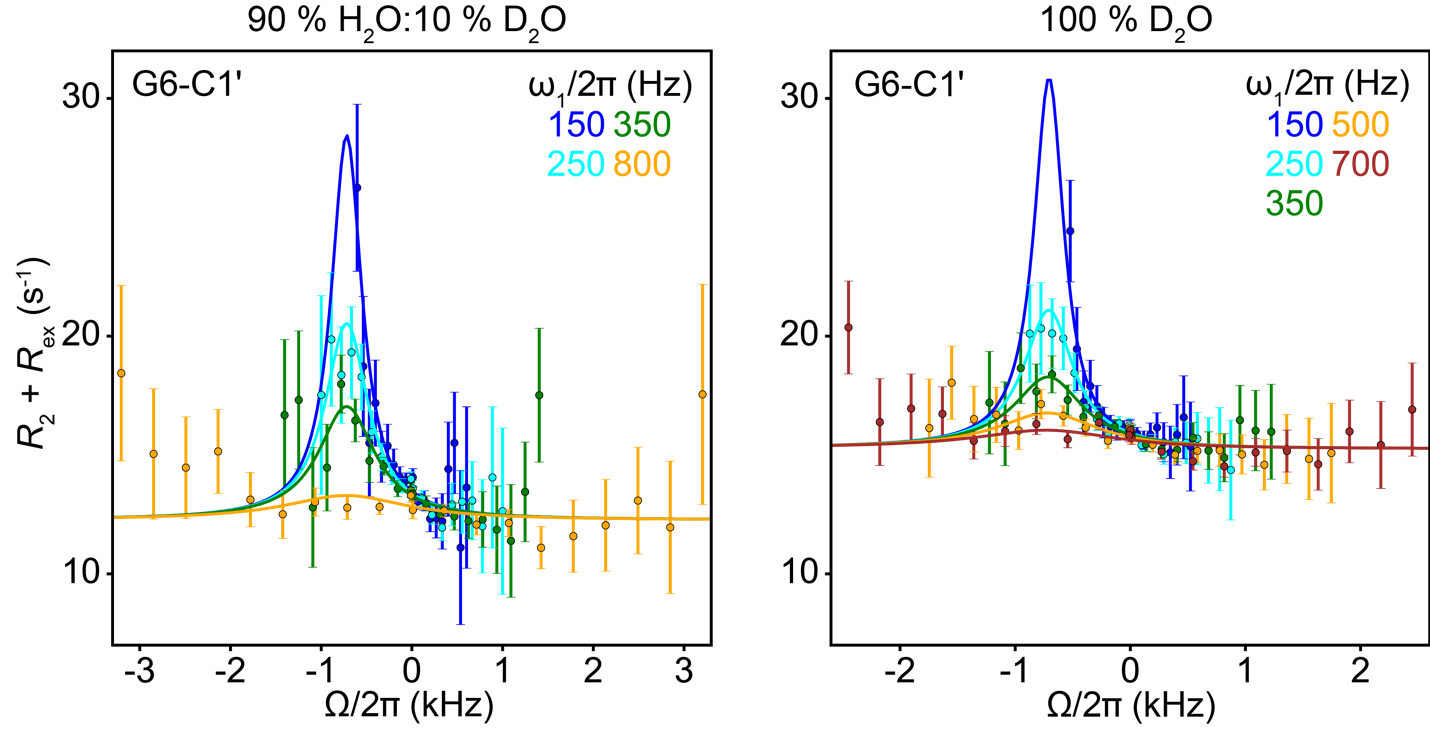


**Supplementary Figure 6**  ^13^C *R*_1ρ_ RD profiles measured for G6-C1' in flGT-CGC. Sample conditions were pH 6.9, 25 °C in 90 % H_2_O:10 % D_2_O or 100 % D_2_O. Experimental data (points) are fit globally to the Bloch-McConnell equations assuming a two-state exchange process with shared exchange parameters together with ^13^C/^15^N *R*_1ρ_ RD data on the atoms of the G•T mismatch which were also fit with shared exchange parameters for the wobble to WC-like exchange of the G•T mismatch assuming a 3-state exchange model in a star-like^1^ topology (Supplementary Figure 9a, Figure 5a, Materials and Methods). Error bars represent the experimental uncertainty in the *R*_1ρ_ data and were computed as described previously by propagating the experimental error in *R*_1ρ_^2^. Spin-lock powers are color-coded


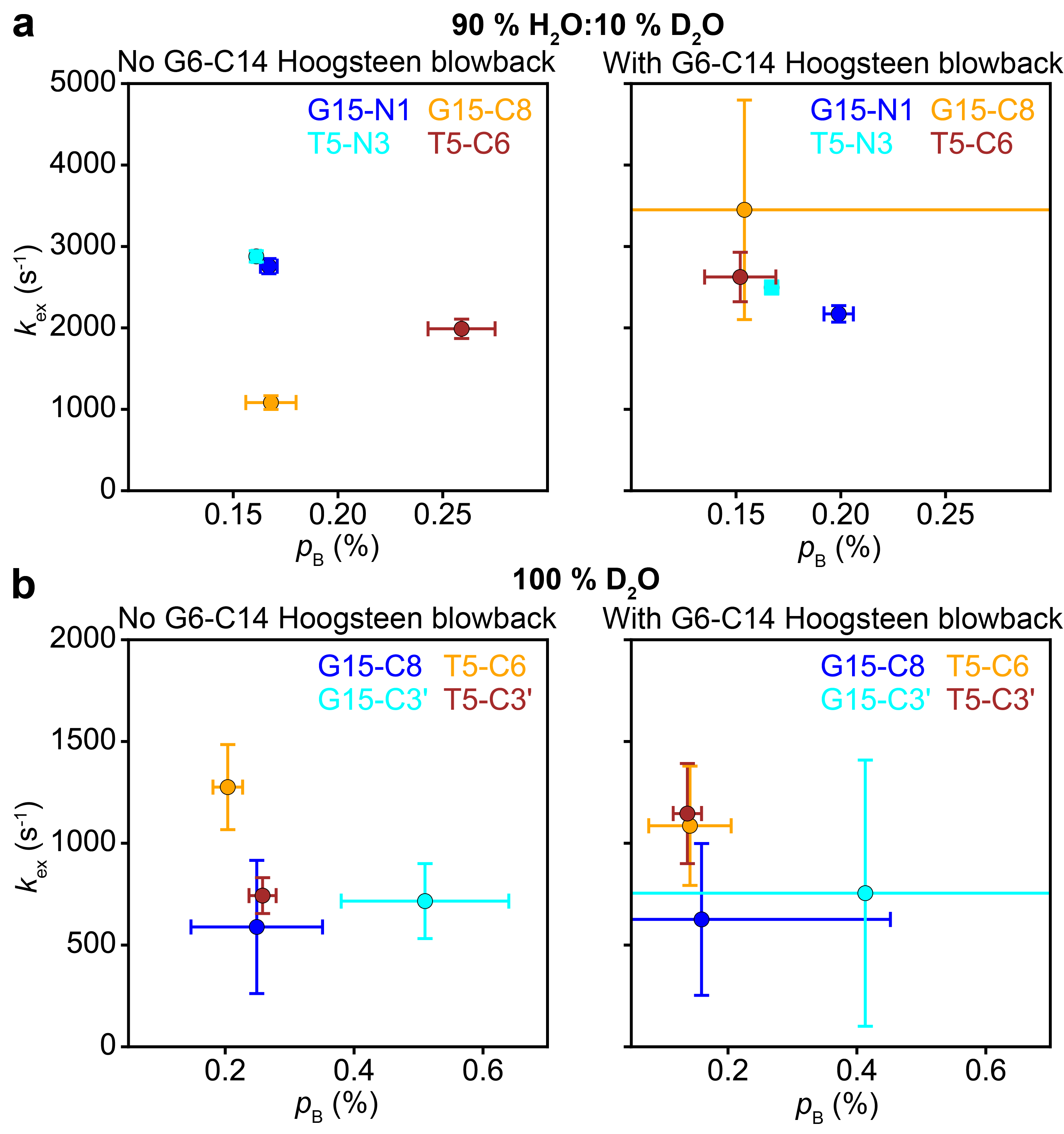


**Supplementary Figure 7** Scatter plots of *k*_ex_ vs. *p*_B_ obtained from individual fitting of the *R*_1ρ_ RD data for the exchange between wobble and WC-like G•T mismatches in slGT-CGC at pH 6.9, 25 °C in (a) 90 % H_2_O: 10% D_2_O and (b) 100 % D_2_O. *R*_1ρ_ profiles were individually fit (Supplementary Tables 3 and 4, Materials and Methods) to the Bloch-McConnell equations without the inclusion of Hoogsteen blowback assuming a 2-state exchange process (No G6-C14 Hoogsteen blowback, left) and with the inclusion of an additional shared exchange contribution due to Hoogsteen blowback of the G6-C14 bp assuming a 3-state exchange process in a star-like exchange topology^1^ (With G6-C14 Hoogsteen blowback, right). Error bars denote errors in fitted parameters computed using a Monte-Carlo scheme as described previously^3^


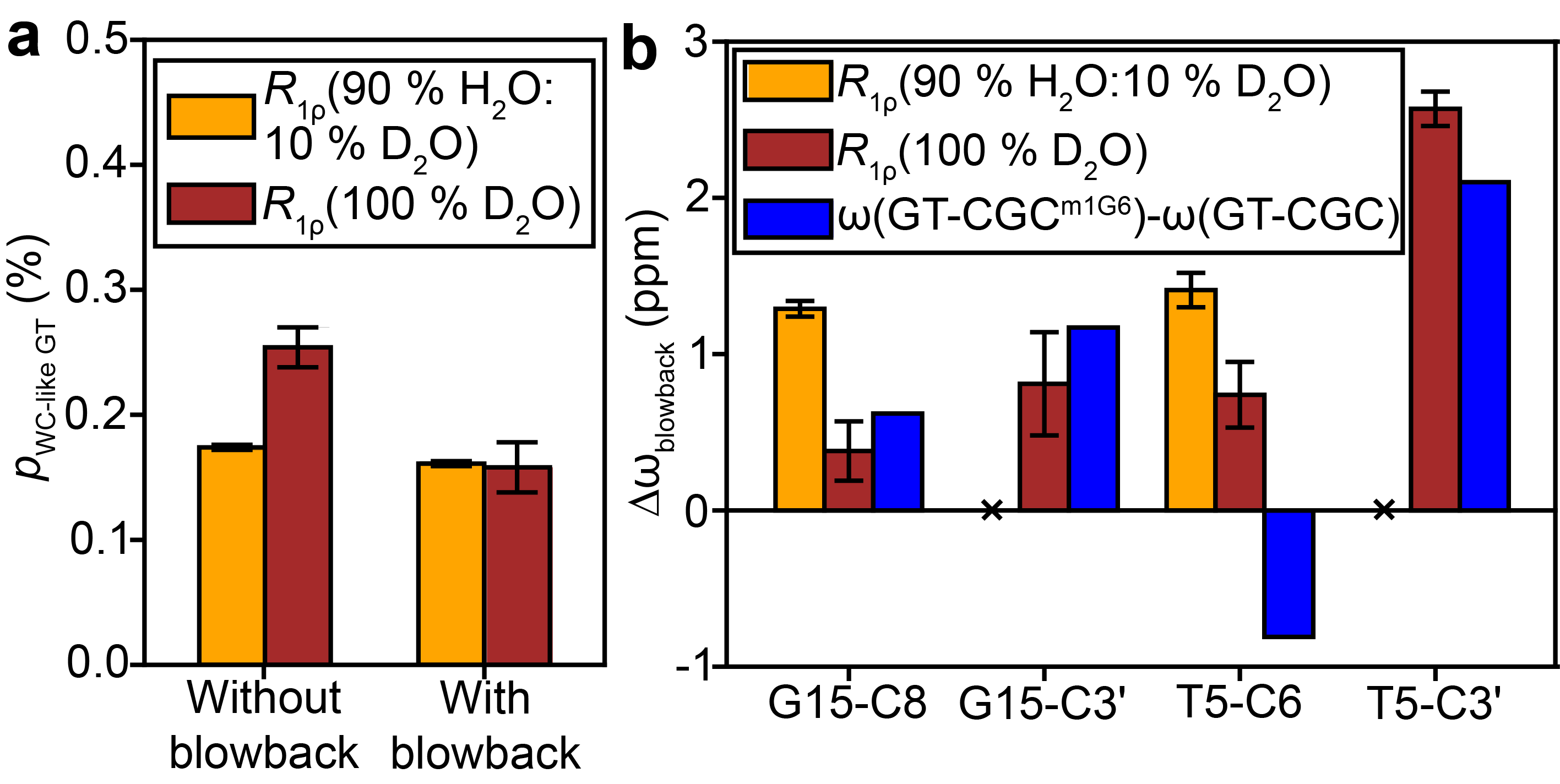


**Supplementary Figure 8** (a) Comparison of the population of the transient tautomeric WC-like G•T mismatch (*p*_WC-like_ _GT_) obtained from global fitting of ^13^C/^15^N *R*_1ρ_ RD data for slGT-CGC in 90 % H_2_O:10 % D_2_O (yellow) and 100 % D_2_O (brown) to the B-M equations with shared exchange parameters, with and without the consideration of an additional shared Hoogsteen blowback exchange contribution (Materials and Methods, Supplementary Tables 3 and 4). (b) Comparison of the Δω for carbon atoms in the G•T mismatch due to Hoogsteen blowback (Δω_blowback_) obtained using ^13^C/^15^N *R*_1ρ_ measurements in 90 % H_2_O:10 %D_2_O (yellow) and 100 % D_2_O (brown), and those estimated as the difference in chemical shifts between GT-CGC^m1G6^ and GT-CGC (blue). The ^13^C/^15^N *R*_1ρ_ RD data was globally fit to the B-M equations with shared exchange parameters for the wobble to WC-like exchange of the G•T mismatch, with the consideration of an additional shared Hoogsteen blowback exchange contribution assuming 3-state exchange in a star-like^1^ topology (Materials and Methods, Supplementary Tables 3 and 4, Supplementary Figure 9a, Figure 5a). ‘X’ denotes resonances for which *R*_1ρ_ was not measured due to interference from the water signal (Materials and Methods). Sample conditions were pH 6.9 and 25 °C


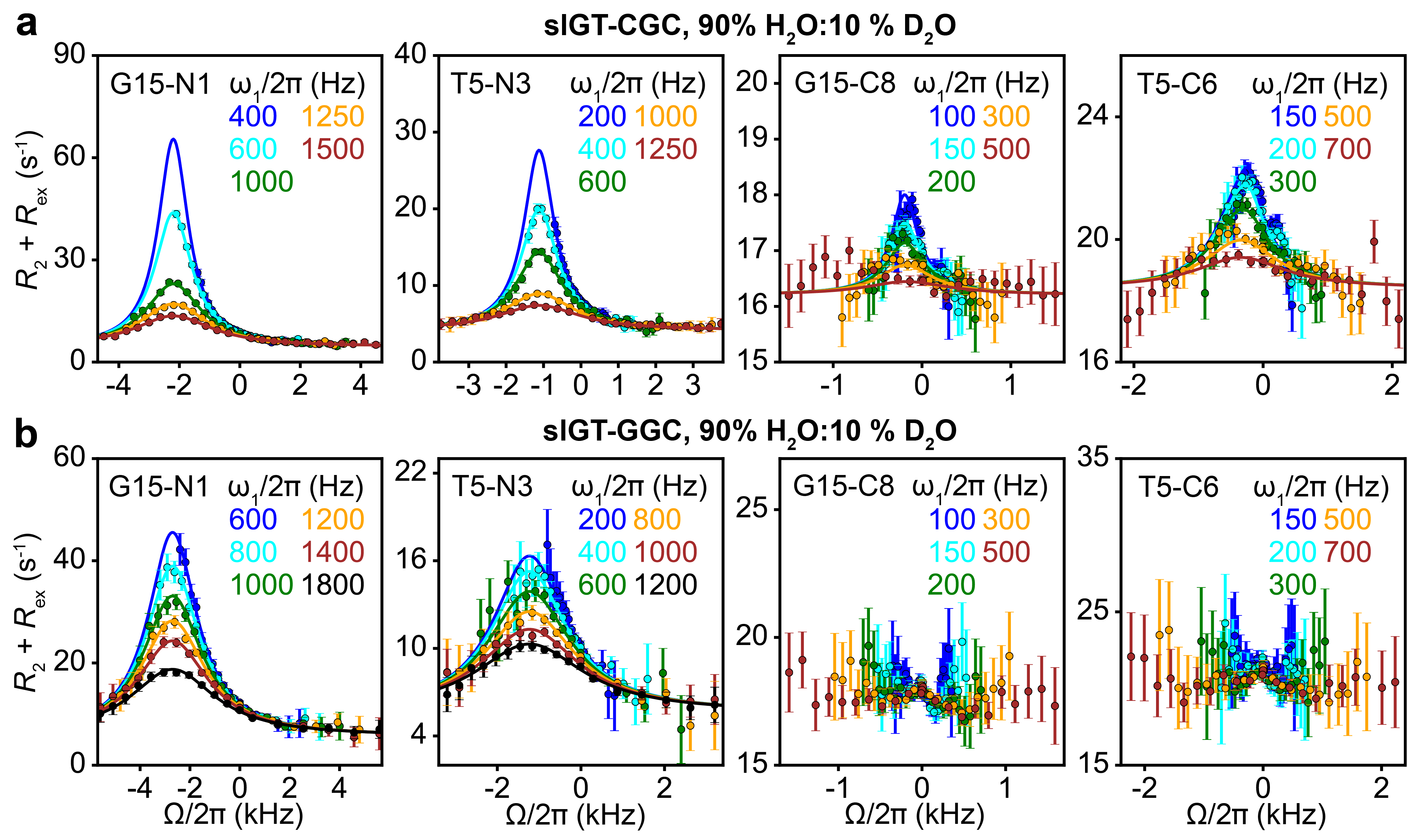


**Supplementary Figure 9** ^13^C and ^15^N *R*_1ρ_ RD profiles measured for base carbon and imino nitrogen atoms in (a) slGT-CGC and (b) slGT-GGC. Sample conditions were pH 6.9, 25 °C in 90 % H_2_O:10 % D_2_O. Experimental data (points) is globally fit to the Bloch-McConnell equations assuming a three state exchange process in a star-like^1^ topology with shared exchange parameters for the wobble to WC-like exchange of the G•T mismatch and the Hoogsteen blowback exchange contribution (Materials and Methods) for slGT-CGC, and a two state exchange process with shared exchange parameters for slGT-GGC. Error bars represent the experimental uncertainty of the *R*_1ρ_ data and were computed as described previously by propagating the experimental error in *R*_1ρ_^2^. Spin-lock powers are color-coded


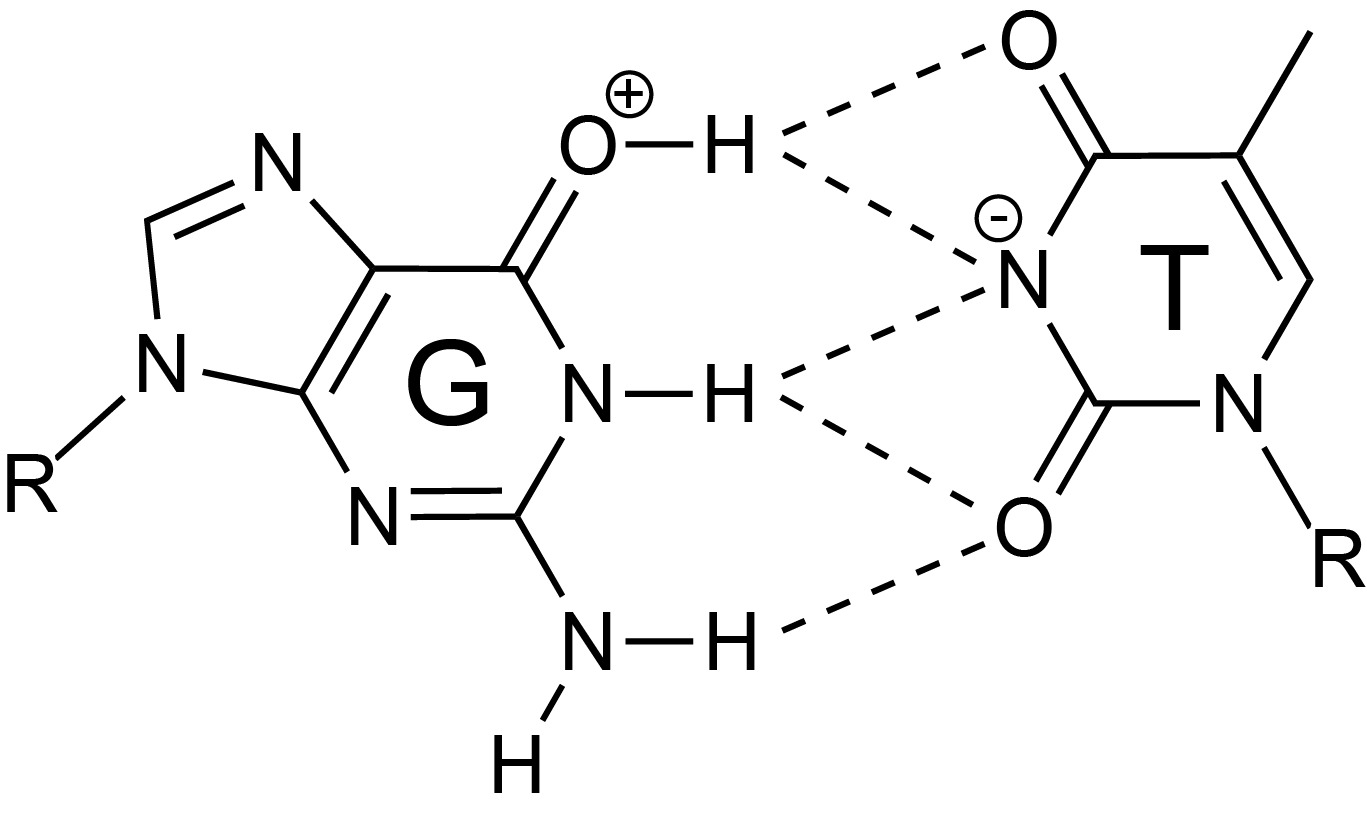


**Supplementary Figure 10** Structure of the transition state for the G•T↔G•T^enol^/G^enol^•T reaction as determined previously by Density Functional Theory (DFT) calculations^4,5^

**
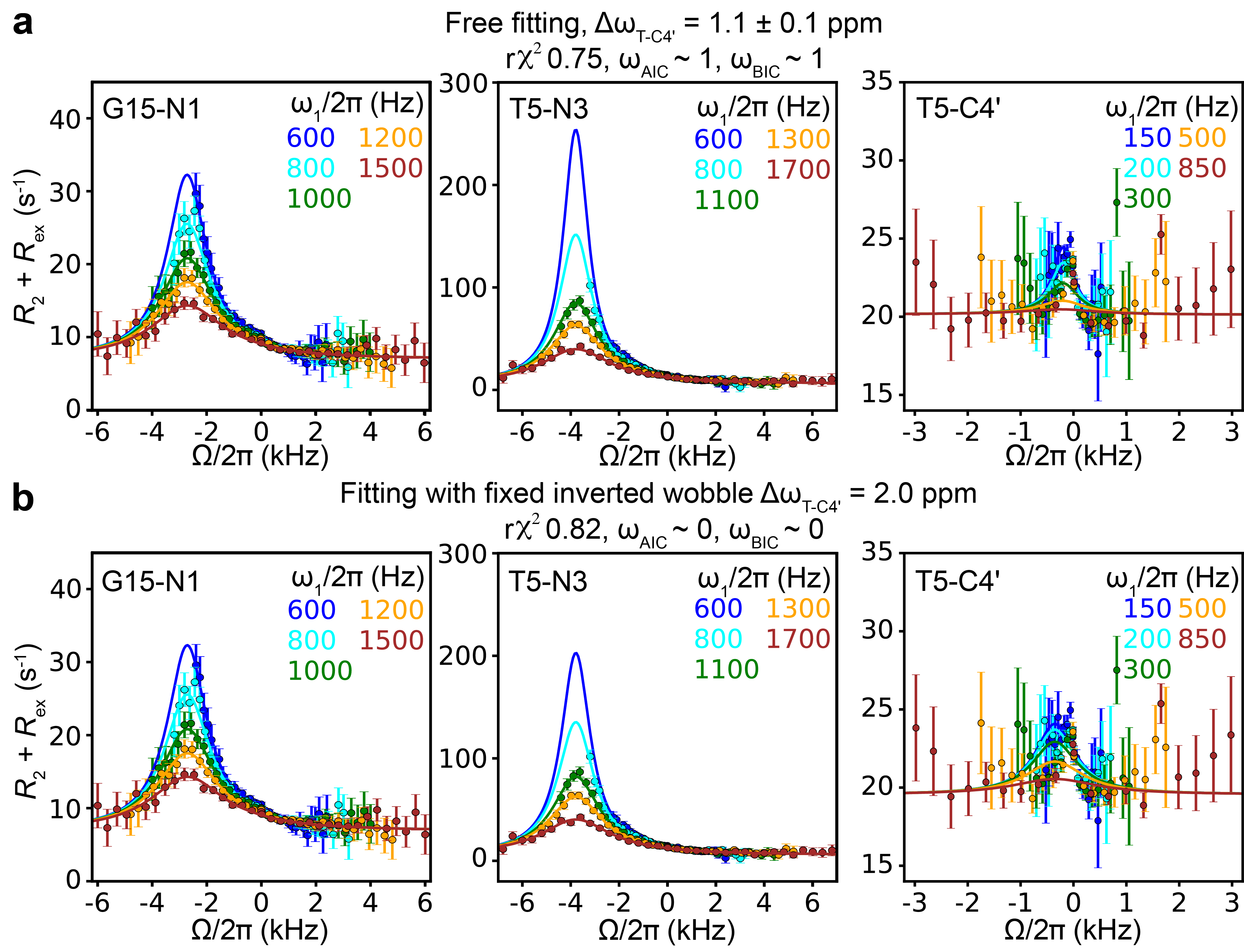
**

**Supplementary Figure 11** Global fitting of the ^13^C and ^15^N *R*_1ρ_ data measured in slGT-GGC (pH 8.8 and 10 °C) to the Bloch-McConnell equations (solid lines) assuming a 3-state exchange process with a star-like^1^ topology and shared exchange parameters (Materials and Methods) with (a) floating Δω_T-C4'_ for the G•T**^¯^** ES and (b) fixing Δω_T-C4'_ = 2.0 ppm expected for the formation of an inverted wobble G•T**^¯^** ES (Figure 7c). The reduced chi square (rχ^2^) obtained on global fitting of the data is indicated along with the statistical Akaike^6^ (ω_AIC_) and Bayesian^7^ information criterion (ω_BIC_) weights. Error bars represent the experimental uncertainty of the *R*_1ρ_ data and were computed as described previously by propagating the experimental error in *R*_1ρ_^2^. Spin-lock powers are color-coded

| PDB ID | Guanine Chain ID:Residue Number | Thymine Chain ID:Residue Number |
| --- | --- | --- |
| 113D | A:4 | B:21 |
| 113D | B:16 | A:9 |
| 1NKC | B:11 | C:32 |
| 3AJK | A:5 | B:20 |
| 3CWS | G:8 | H:18 |
| 3H25 | C:23 | C:11 |
| 3VXV | B:10 | C:4 |
| 3VYQ | B:7 | C:5 |
| 4M3U | T:7 | P:113 |
| 4M3W | T:8 | P:112 |
| 4M3X | T:9 | P:111 |
| 4M42 | P:112 | T:8 |
| 5D9I | Y:9 | X:14 |
| 5DBA | P:4 | T:13 |
| 5HRT | B:2015 | B:2023 |

**Supplementary Table 1** List of G•T mismatches found from the survey of crystal structures in the PDB. Nucleotides are specified by their chain IDs and residue number

| **Nucleus** | **[ω_1_/2π (Hz)] [Ω/2π (Hz)]** |
| --- | --- |
| slGT-CGC (pH 6.9, 25 °C, 90% H_2_O:10% D_2_O) | |
| G15-C8 | [100], [-300, -272, -245, -218, -190, -163, -136, -109, -81, -54, -27, -10, 10, 27, 54, 81, 109, 136, 163, 190, 218, 245, 272, 300]  [150], [-450, -409, -368, -327, -286, -245, -204, -163, -122, -81, -40, -10, 10, 40, 81, 122, 163, 204, 245, 286, 327, 368, 409, 450]  [200], [-600, -545, -490, -436, -381, -327, -272, -218, -163, -109, -54, -10, 10, 54, 109, 163, 218, 272, 327, 381, 436, 490, 545, 600]  [300], [-900, -818, -736, -654, -572, -490, -409, -327, -245, -163, -81, -10, 10, 81, 163, 245, 327, 409, 490, 572, 654, 736, 818, 900]  [500], [-1500, -1363, -1227, -1090, -954, -818, -681, -545, -409, -272, -136, -10, 10, 136, 272, 409, 545, 681, 818, 954, 1090, 1227, 1363, 1500] |
| T5-C6 | [150], [-450, -409, -368, -327, -286, -245, -204, -163, -122, -81, -40, -10, 10, 40, 81, 122, 163, 204, 245, 286, 327, 368, 409, 450]  [200], [-600, -545, -490, -436, -381, -327, -272, -218, -163, -109, -54, -10, 10, 54, 109, 163, 218, 272, 327, 381, 436, 490, 545, 600]  [300], [-900, -818, -736, -654, -572, -490, -409, -327, -245, -163, -81, -10, 10, 81, 163, 245, 327, 409, 490, 572, 654, 736, 818, 900]  [500], [-1500, -1363, -1227, -1090, -954, -818, -681, -545, -409, -272, -136, -10, 10, 136, 272, 409, 545, 681, 818, 954, 1090, 1227, 1363, 1500]  [700], [-2100, -1909, -1718, -1527, -1336, -1145, -954, -763, -572, -381, -190, -10, 10, 190, 381, 572, 763, 954, 1145, 1336, 1527, 1718, 1909, 2100] |
| G15-N1 | [400], [-1400, -1283, -1166, -1050, -933, -816, -700, -583, -466, -350, -233, -116, 116, 233, 350, 466, 583, 700, 816, 933, 1050, 1166, 1283, 1400]  [600], [-2100, -1925, -1750, -1575, -1400, -1225, -1050, -875, -700, -525, -350, -175, 175, 350, 525, 700, 875, 1050, 1225, 1400, 1575, 1750, 1925, 2100]  [1000], [-3500, -3208, -2916, -2625, -2333, -2041, -1750, -1458, -1166, -875, -583, -291, 291, 583, 875, 1166, 1458, 1750, 2041, 2333, 2625, 2916, 3208, 3500]  [1250], [-3750, -3437, -3125, -2812, -2500, -2187, -1875, -1562, -1250, -937, -625, -312, 312, 625, 937, 1250, 1562, 1875, 2187, 2500, 2812, 3125, 3437, 3750]  [1500], [-4500, -4125, -3750, -3375, -3000, -2625, -2250, -1875, -1500, -1125, -750, -375, 375, 750, 1125, 1500, 1875, 2250, 2625, 3000, 3375, 3750, 4125, 4500] |
| T5-N3 | [200], [-700, -641, -583, -525, -466, -408, -350, -291, -233, -175, -116, -58, 58, 116, 175, 233, 291, 350, 408, 466, 525, 583, 641, 700]  [400], [-1400, -1283, -1166, -1050, -933, -816, -700, -583, -466, -350, -233, -116, 116, 233, 350, 466, 583, 700, 816, 933, 1050, 1166, 1283, 1400]  [600], [-2100, -1925, -1750, -1575, -1400, -1225, -1050, -875, -700, -525, -350, -175, 175, 350, 525, 700, 875, 1050, 1225, 1400, 1575, 1750, 1925, 2100]  [1000], [-3500, -3208, -2916, -2625, -2333, -2041, -1750, -1458, -1166, -875, -583, -291, 291, 583, 875, 1166, 1458, 1750, 2041, 2333, 2625, 2916, 3208, 3500]  [1250], [-3750, -3437, -3125, -2812, -2500, -2187, -1875, -1562, -1250, -937, -625, -312, 312, 625, 937, 1250, 1562, 1875, 2187, 2500, 2812, 3125, 3437, 3750] |
| slGT-CGC (pH 6.9, 25 °C, 100% D_2_O) | |
| G15-C3' | [150], [-451, -410, -369, -328, -287, -246, -205, -164, -123, -82, -41, -10, 10, 41, 82, 123, 164, 205, 246, 287, 328, 369, 410, 451]  [200], [-605, -550, -495, -440, -385, -330, -275, -220, -165, -110, -55, -10, 10, 55, 110, 165, 220, 275, 330, 385, 440, 495, 550, 605]  [300], [-902, -820, -738, -656, -574, -492, -410, -328, -246, -164, -82, -10, 10, 82, 164, 246, 328, 410, 492, 574, 656, 738, 820, 902]  [400], [-1199, -1090, -981, -872, -763, -654, -545, -436, -327, -218, -109, -10, 10, 109, 218, 327, 436, 545, 654, 763, 872, 981, 1090, 1199]  [800], [-2398, -2180, -1962, -1744, -1526, -1308, -1090, -872, -654, -436, -218, -10, 10, 218, 436, 654, 872, 1090, 1308, 1526, 1744, 1962, 2180, 2398] |
| G15-C4' | [150], [-451, -410, -369, -328, -287, -246, -205, -164, -123, -82, -41, -10, 10, 41, 82, 123, 164, 205, 246, 287, 328, 369, 410, 451]  [200], [-605, -550, -495, -440, -385, -330, -275, -220, -165, -110, -55, -10, 10, 55, 110, 165, 220, 275, 330, 385, 440, 495, 550, 605]  [300], [-902, -820, -738, -656, -574, -492, -410, -328, -246, -164, -82, -10, 10, 82, 164, 246, 328, 410, 492, 574, 656, 738, 820, 902]  [400], [-1199, -1090, -981, -872, -763, -654, -545, -436, -327, -218, -109, -10, 10, 109, 218, 327, 436, 545, 654, 763, 872, 981, 1090, 1199]  [800], [-2398, -2180, -1962, -1744, -1526, -1308, -1090, -872, -654, -436, -218, -10, 10, 218, 436, 872, 1090, 1308, 1526, 1744, 1962, 2180, 2398] |
| G15-C8 | [150], [-451, -410, -369, -328, -287, -246, -205, -164, -123, -82, -41, -10, 10, 41, 82, 123, 164, 205, 246, 287, 328, 369, 410, 451]  [200], [-605, -550, -495, -440, -385, -330, -275, -220, -165, -110, -55, -10, 10, 55, 110, 165, 220, 275, 330, 385, 440, 495, 550, 605]  [300], [-902, -820, -738, -656, -574, -492, -410, -328, -246, -164, -82, -10, 10, 82, 164, 246, 328, 410, 492, 574, 656, 738, 820, 902]  [400], [-1199, -1090, -981, -872, -763, -654, -545, -436, -327, -218, -109, -10, 10, 109, 218, 327, 436, 545, 654, 763, 872, 981, 1090, 1199]  [800], [-2398, -2180, -1962, -1744, -1526, -1308, -1090, -872, -654, -436, -218, -10, 10, 218, 436, 654, 872, 1090, 1308, 1526, 1744, 1962, 2180, 2398] |
| T5-C3' | [150], [-451, -410, -369, -328, -287, -246, -205, -164, -123, -82, -41, -10, 10, 41, 82, 123, 164, 205, 246, 287, 328, 369, 410, 451]  [200], [-605, -550, -495, -440, -385, -330, -275, -220, -165, -110, -55, -10, 10, 55, 110, 165, 220, 275, 330, 385, 440, 495, 550, 605]  [300], [-902, -820, -738, -656, -574, -492, -410, -328, -246, -164, -82, -10, 10, 82, 164, 246, 328, 410, 492, 574, 656, 738, 820, 902]  [400], [-1199, -1090, -981, -872, -763, -654, -545, -436, -327, -218, -109, -10, 10, 109, 218, 327, 436, 545, 654, 763, 872, 981, 1090, 1199]  [800], [-2398, -2180, -1962, -1744, -1526, -1308, -1090, -654, -436, -218, -10, 10, 218, 436, 654, 872, 1090, 1308, 1526, 1744, 1962, 2180, 2398] |
| T5-C4' | [150], [-451, -410, -369, -287, -246, -205, -164, -123, -82, -41, -10, 10, 41, 82, 123, 164, 205, 246, 287, 328, 369, 410, 451]  [200], [-605, -550, -495, -440, -385, -330, -275, -220, -165, -110, -55, -10, 10, 55, 110, 165, 220, 275, 330, 385, 440, 495, 550, 605]  [300], [-902, -820, -738, -656, -574, -492, -410, -328, -246, -164, -82, -10, 10, 82, 164, 246, 328, 410, 492, 574, 656, 738, 820, 902]  [400], [-1199, -1090, -981, -872, -763, -654, -545, -436, -327, -218, -109, -10, 10, 109, 218, 327, 436, 545, 654, 763, 872, 981, 1090, 1199]  [800], [-2398, -2180, -1962, -1744, -1526, -1308, -1090, -872, -654, -436, -218, -10, 10, 218, 436, 654, 1090, 1308, 1526, 1744, 1962, 2180, 2398] |
| T5-C6 | [150], [-451, -410, -369, -328, -287, -246, -205, -164, -123, -82, -41, -10, 10, 41, 82, 123, 164, 205, 246, 287, 328, 369, 410, 451]  [200], [-605, -550, -495, -440, -385, -330, -275, -220, -165, -110, -55, -10, 10, 55, 110, 165, 220, 275, 330, 385, 440, 495, 550, 605]  [300], [-902, -820, -738, -656, -574, -492, -410, -328, -246, -164, -82, -10, 10, 82, 164, 246, 328, 410, 492, 574, 656, 738, 820, 902]  [400], [-1199, -1090, -981, -872, -763, -654, -545, -436, -327, -218, -109, -10, 10, 109, 218, 327, 436, 545, 654, 763, 872, 981, 1090, 1199]  [800], [-2398, -2180, -1962, -1744, -1526, -1308, -1090, -872, -654, -436, -218, -10, 10, 218, 436, 654, 872, 1090, 1308, 1526, 1744, 1962, 2180, 2398] |
| flGT-CGC (pH 6.9, 25 °C, 100% D_2_O) | |
| G6-C1' | [150], [-522, -464, -406, -348, -290, -232, -174, -116, -58, -10, 10, 58, 116, 174, 232, 290, 348, 406, 464, 522]  [250], [-873, -776, -679, -582, -485, -388, -291, -194, -97, -10, 10, 97, 194, 291, 388, 485, 582, 679, 776, 873]  [350], [-1224, -1088, -952, -816, -680, -544, -408, -272, -136, -10, 10, 136, 272, 408, 544, 680, 816, 952, 1088, 1224]  [500], [-1746, -1552, -1358, -1164, -970, -776, -582, -388, -194, -10, 10, 194, 388, 582, 776, 970, 1164, 1358, 1552, 1746]  [700], [-2448, -2176, -1904, -1632, -1360, -1088, -816, -544, -272, -10, 10, 272, 544, 816, 1088, 1360, 1632, 1904, 2176, 2448] |
| flGT-CGC (pH 6.9, 25 °C, 90% H_2_O: 10 % D_2_O) | |
| G6-C1' | [150.0], [-603.0, -536, -469, -402, -335, -268, -201, -134, -67, -10, 10, 67, 134, 201, 268, 335, 402, 469, 536, 603]  [250.0], [-999.0, -888, -777, -666, -555, -444, -333, -222, -111, -10, 10, 111, 222, 333, 444, 555, 666, 777, 888, 999]  [350.0], [-1404.0, -1248, -1092, -936, -780, -624, -468, -312, -156, -10, 10, 156, 312, 468, 624, 780, 936, 1092, 1248, 1404]  [800.0], [-3204.0, -2848, -2492, -2136, -1780, -1424, -1068, -712, -356, -10, 10, 356, 712, 1068, 1424, 1780, 2136, 2492, 2848, 3204] |
| slGT-GGC (pH 6.9, 25 °C, 90% H_2_O:10% D_2_O) | |
| G15-N1 | [600], [-2398, -2180, -1962, -1744, -1526, -1308, -1090, -872, -654, -436, -218, -10, 218, 654, 1090, 1526, 1962, 2398]  [800], [-3201, -2910, -2619, -2328, -2037, -1746, -1455, -1164, -873, -582, -291, -10, 291, 873, 1455, 2037, 2619, 3201]  [1000], [-4004, -3640, -3276, -2912, -2548, -2184, -1820, -1456, -1092, -728, -364, -10, 364, 1092, 1820, 2548, 3276, 4004]  [1200], [-4796, -4360, -3924, -3488, -3052, -2616, -2180, -1744, -1308, -872, -436, -10, 436, 1308, 2180, 3052, 3924, 4796]  [1400], [-5599, -5090, -4581, -4072, -3563, -3054, -2545, -2036, -1527, -1018, -509, -10, 509, 1527, 2545, 3563, 4581, 5599]  [1800], [-5599, -5090, -4581, -4072, -3563, -3054, -2545, -2036, -1527, -1018, -509, -10, 509, 1527, 2545, 3563, 4581, 5599] |
| T5-N3 | [200], [-803, -730, -657, -584, -511, -438, -365, -292, -219, -146, -73, -10, 73, 219, 365, 511, 657, 803]  [400], [-1595, -1450, -1305, -1160, -1015, -870, -725, -580, -435, -290, -145, -10, 145, 435, 725, 1015, 1305, 1595]  [600], [-2398, -2180, -1962, -1744, -1526, -1308, -1090, -872, -654, -436, -218, -10, 218, 654, 1090, 1526, 1962, 2398]  [800], [-3201, -2910, -2619, -2328, -2037, -1746, -1455, -1164, -873, -582, -291, -10, 291, 873, 1455, 2037, 2619, 3201]  [1000], [-3201, -2910, -2619, -2328, -2037, -1746, -1455, -1164, -873, -582, -291, -10, 291, 873, 1455, 2037, 2619, 3201]  [1200], [-3201, -2910, -2619, -2328, -2037, -1746, -1455, -1164, -873, -582, -291, -10, 291, 873, 1455, 2037, 2619, 3201] |
| G15-C8 | [100], [-352, -320, -288, -256, -224, -192, -160, -128, -96, -64, -32, -10, 10, 32, 64, 96, 128, 160, 192, 224, 256, 288, 320, 352]  [150], [-528, -480, -432, -384, -336, -288, -240, -192, -144, -96, -48, -10, 10, 48, 96, 144, 192, 240, 288, 336, 384, 432, 480, 528]  [200], [-704, -640, -576, -512, -448, -384, -320, -256, -192, -128, -64, -10, 10, 64, 128, 192, 256, 320, 384, 448, 512, 576, 640, 704]  [300], [-1045, -950, -855, -760, -665, -570, -475, -380, -285, -190, -95, -10, 10, 95, 190, 285, 380, 475, 570, 665, 760, 855, 950, 1045]  [500], [-1749, -1590, -1431, -1272, -1113, -954, -795, -636, -477, -318, -159, -10, 10, 159, 318, 477, 636, 795, 954, 1113, 1272, 1431, 1590, 1749] |
| T5-C6 | [150], [-528, -480, -432, -384, -336, -288, -240, -192, -144, -96, -48, -10, 10, 48, 96, 144, 192, 240, 288, 336, 384, 432, 480, 528]  [200], [-704, -640, -576, -512, -448, -384, -320, -256, -192, -128, -64, -10, 10, 64, 128, 192, 256, 320, 384, 448, 512, 576, 640, 704]  [300], [-1045, -950, -855, -760, -665, -570, -475, -380, -285, -190, -95, -10, 10, 95, 190, 285, 380, 475, 570, 665, 760, 855, 950, 1045]  [500], [-1749, -1590, -1431, -1272, -1113, -954, -795, -636, -477, -318, -159, -10, 10, 159, 318, 477, 636, 795, 954, 1113, 1272, 1431, 1590, 1749]  [700], [-2453, -2230, -2007, -1784, -1561, -1338, -1115, -892, -669, -446, -223, -10, 10, 223, 446, 669, 892, 1115, 1338, 1561, 1784, 2007, 2230, 2453] |
| slGT-GGC (pH 6.9, 25 °C, 100% D_2_O) | |
| G15-C3' | [150], [-522, -464, -406, -348, -290, -232, -174, -116, -58, -10, 10, 58, 116, 174, 232, 290, 348, 406, 464, 522]  [200], [-702, -624, -546, -468, -390, -312, -234, -156, -78, -10, 10, 78, 156, 234, 312, 390, 468, 546, 624, 702]  [250], [-873, -776, -679, -582, -485, -388, -291, -194, -97, -10, 10, 97, 194, 291, 388, 485, 582, 679, 776, 873]  [300], [-1053, -936, -819, -702, -585, -468, -351, -234, -117, -10, 10, 117, 234, 351, 468, 585, 702, 819, 936, 1053]  [400], [-1404, -1248, -1092, -936, -780, -624, -468, -312, -156, -10, 10, 156, 312, 468, 624, 780, 936, 1092, 1248, 1404] |
| G15-C4' | [150], [-522, -464, -406, -348, -290, -232, -174, -116, -58, -10, 10, 58, 116, 174, 232, 290, 348, 406, 464, 522]  [200], [-702, -624, -546, -468, -390, -312, -234, -156, -78, -10, 10, 78, 156, 234, 312, 390, 468, 546, 624, 702]  [250], [-873, -776, -679, -582, -485, -388, -291, -194, -97, -10, 10, 97, 194, 291, 388, 485, 582, 679, 776, 873]  [300], [-1053, -936, -819, -702, -585, -468, -351, -234, -117, -10, 10, 117, 234, 351, 468, 585, 702, 819, 936, 1053]  [400], [-1404, -1248, -1092, -936, -780, -624, -468, -312, -156, -10, 10, 156, 312, 468, 624, 780, 936, 1092, 1248, 1404] |
| G15-C8 | [150], [-522, -464, -406, -348, -290, -232, -174, -116, -58, -10, 10, 58, 116, 174, 232, 290, 348, 406, 464, 522]  [200], [-702, -624, -546, -468, -390, -312, -234, -156, -78, -10, 10, 78, 156, 234, 312, 390, 468, 546, 624, 702]  [250], [-873, -776, -679, -582, -485, -388, -291, -194, -97, -10, 10, 97, 194, 291, 388, 485, 582, 679, 776, 873]  [300], [-1053, -936, -819, -702, -585, -468, -351, -234, -117, -10, 10, 117, 234, 351, 468, 585, 702, 819, 936, 1053]  [400], [-1404, -1248, -1092, -936, -780, -624, -468, -312, -156, -10, 10, 156, 312, 468, 624, 780, 936, 1092, 1248, 1404] |
| T5-C3' | [150], [-522, -464, -406, -348, -290, -232, -174, -116, -58, -10, 10, 58, 116, 174, 232, 290, 348, 406, 464, 522]  [200], [-702, -624, -546, -468, -390, -312, -234, -156, -78, -10, 10, 78, 156, 234, 312, 390, 468, 546, 624, 702]  [250], [-873, -776, -679, -582, -485, -388, -291, -194, -97, -10, 10, 97, 194, 291, 388, 485, 582, 679, 776, 873]  [350], [-1224, -1088, -952, -816, -680, -544, -408, -272, -136, -10, 10, 136, 272, 408, 544, 680, 816, 952, 1088, 1224]  [800], [-2799, -2488, -2177, -1866, -1555, -1244, -933, -622, -311, -10, 10, 311, 622, 933, 1244, 1555, 1866, 2177, 2488, 2799] |
| T5-C4' | [150], [-522, -464, -406, -348, -290, -232, -174, -116, -58, -10, 10, 58, 116, 174, 232, 290, 348, 406, 464, 522]  [200], [-702, -624, -546, -468, -390, -312, -234, -156, -78, -10, 10, 78, 156, 234, 312, 390, 468, 546, 624, 702]  [250], [-873, -776, -679, -582, -485, -388, -291, -194, -97, -10, 10, 97, 194, 291, 388, 485, 582, 679, 776, 873]  [300], [-1053, -936, -819, -702, -585, -468, -351, -234, -117, -10, 10, 117, 234, 351, 468, 585, 702, 819, 936, 1053]  [400], [-1404, -1248, -1092, -936, -780, -624, -468, -312, -156, -10, 10, 156, 312, 468, 624, 780, 936, 1092, 1248, 1404] |
| T5-C6 | [150], [-522, -464, -406, -348, -290, -232, -174, -116, -58, -10, 10, 58, 116, 174, 232, 290, 348, 406, 464, 522]  [200], [-702, -624, -546, -468, -390, -312, -234, -156, -78, -10, 10, 78, 156, 234, 312, 390, 468, 546, 624, 702]  [250], [-873, -776, -679, -582, -485, -388, -291, -194, -97, -10, 10, 97, 194, 291, 388, 485, 582, 679, 776, 873]  [300], [-1053, -936, -819, -702, -585, -468, -351, -234, -117, -10, 10, 117, 234, 351, 468, 585, 702, 819, 936, 1053]  [600], [-2097, -1864, -1631, -1398, -1165, -932, -699, -466, -233, -10, 10, 233, 466, 699, 932, 1165, 1398, 1631, 1864, 2097] |
| slGT-GGC (pH 8.8, 10 °C, 90% H_2_O: 10% D_2_O) | |
| G15-N1 | [600], [-2397, -2256, -2115, -1974, -1833, -1692, -1551, -1410, -1269, -1128, -987, -846, -705, -564, -423, -282, -141, -10, 10, 141, 282, 423, 564, 705, 846, 987, 1128, 1269, 1410, 1551, 1692, 1833, 1974, 2115, 2256, 2397]  [800], [-3196, -3008, -2820, -2632, -2444, -2256, -2068, -1880, -1692, -1504, -1316, -1128, -940, -752, -564, -376, -188, -10, 10, 188, 376, 564, 752, 940, 1128, 1316, 1504, 1692, 1880, 2068, 2256, 2444, 2632, 2820, 3008, 3196]  [1000], [-3995, -3760, -3525, -3290, -3055, -2820, -2585, -2350, -2115, -1880, -1645, -1410, -1175, -940, -705, -470, -235, -10, 10, 235, 470, 705, 940, 1175, 1410, 1645, 1880, 2115, 2350, 2585, 2820, 3055, 3290, 3525, 3760, 3995]  [1200], [-4794, -4512, -4230, -3948, -3666, -3384, -3102, -2820, -2538, -2256, -1974, -1692, -1410, -1128, -846, -564, -282, -10, 10, 282, 564, 846, 1128, 1410, 1692, 1974, 2256, 2538, 2820, 3102, 3384, 3666, 3948, 4230, 4512, 4794]  [1500], [-6001, -5648, -5295, -4942, -4589, -4236, -3883, -3530, -3177, -2824, -2471, -2118, -1765, -1412, -1059, -706, -353, -10, 10, 353, 706, 1059, 1412, 1765, 2118, 2471, 2824, 3177, 3530, 3883, 4236, 4589, 4942, 5295, 5648, 6001] |
| T5-N3 | [600], [-2397, -2256, -2115, -1974, -1833, -1692, -1551, -1410, -1269, -1128, -987, -846, -705, -564, -423, -282, -141, -10, 10, 141, 282, 423, 564, 705, 846, 987, 1128, 1269, 1410, 1551, 1692, 1833, 1974, 2115, 2256, 2397]  [800], [-3196, -3008, -2820, -2632, -2444, -2256, -2068, -1880, -1692, -1504, -1316, -1128, -940, -752, -564, -376, -188, -10, 10, 188, 376, 564, 752, 940, 1128, 1316, 1504, 1692, 1880, 2068, 2256, 2444, 2632, 2820, 3008, 3196]  [1100], [-4403, -4144, -3885, -3626, -3367, -3108, -2849, -2590, -2331, -2072, -1813, -1554, -1295, -1036, -777, -518, -259, -10, 10, 259, 518, 777, 1036, 1295, 1554, 1813, 2072, 2331, 2590, 2849, 3108, 3367, 3626, 3885, 4144, 4403]  [1300], [-5202, -4896, -4590, -4284, -3978, -3672, -3366, -3060, -2754, -2448, -2142, -1836, -1530, -1224, -918, -612, -306, -10, 10, 306, 612, 918, 1224, 1530, 1836, 2142, 2448, 2754, 3060, 3366, 3672, 3978, 4284, 4590, 4896, 5202]  [1700], [-6800, -6400, -6000, -5600, -5200, -4800, -4400, -4000, -3600, -3200, -2800, -2400, -2000, -1600, -1200, -800, -400, -10, 10, 400, 800, 1200, 1600, 2000, 2400, 2800, 3200, 3600, 4000, 4400, 4800, 5200, 5600, 6000, 6400, 6800] |
| T5-C4' | [150], [-522, -464, -406, -348, -290, -232, -174, -116, -58, -10, 10, 58, 116, 174, 232, 290, 348, 406, 464, 522]  [200], [-702, -624, -546, -468, -390, -312, -234, -156, -78, -10, 10, 78, 156, 234, 312, 390, 468, 546, 624, 702]  [300], [-1053, -936, -819, -702, -585, -468, -351, -234, -117, -10, 10, 117, 234, 351, 468, 585, 702, 819, 936, 1053]  [500], [-1746, -1552, -1358, -1164, -970, -776, -582, -388, -194, -10, 10, 194, 388, 582, 776, 970, 1164, 1358, 1552, 1746]  [850], [-2979, -2648, -2317, -1986, -1655, -1324, -993, -662, -331, -10, 10, 331, 662, 993, 1324, 1655, 1986, 2317, 2648, 2979] |

**Supplementary Table 2** List of spin-lock powers (ω_1_/2π, in Hz) and offsets (Ω/2π, in Hz) used in off-resonance ^13^C/^15^N *R*_1ρ_ experiments

|  | Parameter | G15-N1 | T5-N3 | G15-C8 | T5-C6 | G6-C1' |
| --- | --- | --- | --- | --- | --- | --- |
| Individual  Fitting | *p*_B_ (%) | 0.167 ± 0.004 | 0.161 ± 0.002 | 0.168 ± 0.012 | 0.259 ± 0.016 | 0.101 ± 0.009 |
|  | *k*_exAB_ (s^-1^) | 2758 ± 93 | 2878 ± 69 | 1083 ± 84 | 1989 ± 119 | 1732 ± 369 |
|  | Δω_AB_ (ppm) | 36.08 ± 0.12 | 18.36 ± 0.10 | 1.26 ± 0.06 | 2.04 ± 0.08 | 4.12 ± 0.21 |
|  | *R*_1_ (s^-1^) | 2.43 ± 0.01 | 2.25 ± 0.01 | 3.00 ± 0.01 | 3.67 ± 0.02 | 1.74 ± 0.03 |
|  | *R*_2_ (s^-1^) | 4.57 ± 0.04 | 4.16 ± 0.04 | 16.21 ± 0.02 | 18.54 ± 0.06 | 12.02 ± 0.17 |
|  | Red. χ^2^ | 0.81 | 0.63 | 0.86 | 1.10 | 0.69 |
| Individual Fitting with shared G6-C14 Hoogsteen  Blowback | *p*_B_ (%) | 0.199 ± 0.007 | 0.167 ± 0.003 | 0.154 ± 0.758 | 0.152 ± 0.017 | - |
|  | *p_C_* (%) | 0.154 ± 0.013 | | | | |
|  | *k*_exAB_ (s^-1^) | 2172 ± 101 | 2496 ± 81 | 3450 ± 1348 | 2625 ± 304 | - |
|  | *k*_exAC_ (s^-1^) | 798 ± 92 | | | | |
|  | Δω_AB_ (ppm) | 36.26 ± 0.12 | 18.42 ± 0.11 | 0.70 ± 1.81 | 2.51 ± 0.14 | - |
|  | Δω_AC_ (ppm) | -2.04 ± 0.24 | 1.50 ± 0.23 | 1.23 ± 0.07 | 1.29 ± 0.20 | 4.09 ± 0.16 |
|  | *R*_1_ (s^-1^) | 2.42 ± 0.01 | 2.24 ± 0.01 | 3.00 ± 0.01 | 3.67 ± 0.02 | 1.70 ± 0.03 |
|  | *R*_2_ (s^-1^) | 4.69 ± 0.04 | 4.31 ± 0.04 | 16.19 ± 0.04 | 18.50 ± 0.06 | 12.30 ± 0.30 |
|  | Red. χ^2^ | 0.82 | | | | |
| Global Fitting without G6-C14 Hoogsteen  Blowback | *p*_B_ (%) | 0.174 ± 0.002 | | | | - |
|  | *k*_exAB_ (s^-1^) | 2545 ± 51 | | | | - |
|  | Δω_AB_ (ppm) | 36.14 ± 0.15 | 18.11 ± 0.12 | 1.52 ± 0.04 | 2.71 ± 0.06 | - |
|  | *R*_1_ (s^-1^) | 2.43 ± 0.01 | 2.23 ± 0.01 | 3.01 ± 0.01 | 3.64 ± 0.02 | - |
|  | *R*_2_ (s^-1^) | 4.68 ± 0.04 | 4.23 ± 0.03 | 15.98 ± 0.03 | 18.50 ± 0.04 | - |
|  | Red. χ^2^ | 1.28 | | | | - |
| Global Fitting with shared G6-C14 Hoogsteen  Blowback | *p*_B_ (%) | 0.161 ± 0.002 | | | | - |
|  | *p_C_* (%) | 0.151 ± 0.009 | | | | |
|  | *k*_exAB_ (s^-1^) | 2844 ± 49 | | | | - |
|  | *k*_exAC_ (s^-1^) | 867 ± 72 | | | | |
|  | Δω_AB_ (ppm) | 36.18 ± 0.12 | 18.41 ± 0.11 | 0.50 ± 0.12 | 2.45 ± 0.09 | - |
|  | Δω_AC_ (ppm) | -1.92 ± 0.22 | 1.07 ± 0.23 | 1.29 ± 0.05 | 1.41 ± 0.11 | 4.08 ± 0.15 |
|  | *R*_1_ (s^-1^) | 2.45 ± 0.01 | 2.25 ± 0.01 | 3.00 ± 0.01 | 3.67 ± 0.01 | 1.70 ± 0.03 |
|  | *R*_2_ (s^-1^) | 4.52 ± 0.03 | 4.14 ± 0.03 | 16.21 ± 0.03 | 18.41 ± 0.04 | 12.27 ± 0.10 |
|  | Red. χ^2^ | 0.79 | | | | |

**Supplementary Table 3** Summary of exchange parameters obtained from fitting ^13^C and ^15^N *R*_1ρ_ data measured on slGT-CGC at pH 6.9 and 25 °C in 90 % H_2_O:10 % D_2_O. State B corresponds to the tautomeric WC-like G•T mismatch. Hoogsteen blowback refers to the passive sensing of the Watson-Crick to Hoogsteen exchange of the G6-C14 bp (state C) by the atoms of the G•T mismatch. Red. ${}^{2}$ denotes the reduced ${}^{2}$ obtained on fitting the *R*_1ρ_ data

|  | Parameter | G15-C8 | G15-C3' | T5-C6 | T5-C3' | G6-C1' |
| --- | --- | --- | --- | --- | --- | --- |
| Individual  Fitting | *p*_B_ (%) | 0.249 ± 0.102 | 0.510 ± 0.130 | 0.204 ± 0.023 | 0.258 ± 0.021 | 0.140 ± 0.146 |
|  | *k*_exAB_ (s^-1^) | 589 ± 327 | 716 ± 184 | 1276 ± 209 | 743 ± 88 | 488 ± 552 |
|  | Δω_AB_ (ppm) | 1.11 ± 0.13 | 0.70 ± 0.08 | 1.73 ± 0.13 | 2.21 ± 0.05 | 4.08 ± 0.20 |
|  | *R*_1_ (s^-1^) | 2.12 ± 0.02 | 1.76 ± 0.02 | 2.54 ± 0.03 | 1.58 ± 0.02 | 1.71 ± 0.03 |
|  | *R*_2_ (s^-1^) | 20.93 ± 0.05 | 12.97 ± 0.04 | 24.30 ± 0.08 | 11.73 ± 0.04 | 15.33 ± 0.06 |
|  | Red. χ^2^ | 1.84 | 1.46 | 0.90 | 1.37 | 0.96 |
| Individual Fitting with shared  G6-C14 Hoogsteen  Blowback | *p*_B_ (%) | 0.159 ± 0.293 | 0.413 ± 0.857 | 0.141 ± 0.064 | 0.137 ± 0.022 | - |
|  | *p_C_* (%) | 0.365 ± 0.229 | | | | |
|  | *k*_exAB_ (s^-1^) | 626 ± 373 | 755 ± 654 | 1086 ± 293 | 1146 ± 246 | - |
|  | *k*_exAC_ (s^-1^) | 201 ± 127 | | | | |
|  | Δω_AB_ (ppm) | 1.27 ± 0.54 | 0.74 ± 0.21 | 2.10 ± 0.34 | 1.73 ± 0.17 | - |
|  | Δω_AC_ (ppm) | 0.56 ± 1.33 | 0.39 ± 2.41 | 0.78 ± 0.61 | 2.49 ± 0.14 | 4.00 ± 0.19 |
|  | *R*_1_ (s^-1^) | 2.12 ± 0.02 | 1.76 ± 0.02 | 2.52 ± 0.04 | 1.60 ± 0.02 | 1.70 ± 0.03 |
|  | *R*_2_ (s^-1^) | 20.93 ± 0.05 | 12.97 ± 0.04 | 24.38 ± 0.10 | 11.70 ± 0.05 | 15.27 ± 0.07 |
|  | Red. χ^2^ | 1.30 | | | | |
| Global fitting without G6-C14 Hoogsteen blowback | *p*_B_ (%) | 0.254 ± 0.016 | | | | - |
|  | *k*_exAB_ (s^-1^) | 776 ± 73 | | | | - |
|  | Δω_AB_ (ppm) | 1.01 ± 0.05 | 1.01 ± 0.04 | 1.66 ± 0.08 | 2.20 ± 0.05 | - |
|  | *R*_1_ (s^-1^) | 2.13 ± 0.02 | 1.74 ± 0.02 | 2.53 ± 0.04 | 1.59 ± 0.02 | - |
|  | *R*_2_ (s^-1^) | 20.90 ± 0.04 | 13.00 ± 0.03 | 24.43 ± 0.07 | 11.72 ± 0.04 | - |
|  | Red. χ^2^ | 1.43 | | | | - |
| Global Fitting with shared  G6-C14 Hoogsteen  Blowback | *p*_B_ (%) | 0.158 ± 0.020 | | | | - |
|  | *p_C_* (%) | 0.361 ± 0.217 | | | | |
|  | *k*_exAB_ (s^-1^) | 909 ± 142 | | | | - |
|  | *k*_exAC_ (s^-1^) | 205 ± 125 | | | | |
|  | Δω_AB_ (ppm) | 1.24 ± 0.09 | 0.98 ± 0.24 | 2.07 ± 0.14 | 1.64 ± 0.13 | - |
|  | Δω_AC_ (ppm) | 0.38 ± 0.19 | 0.81 ± 0.33 | 0.74 ± 0.21 | 2.57 ± 0.11 | 4.00 ± 0.19 |
|  | *R*_1_ (s^-1^) | 2.12 ± 0.02 | 1.75 ± 0.02 | 2.51 ± 0.04 | 1.59 ± 0.02 | 1.70 ± 0.03 |
|  | *R*_2_ (s^-1^) | 20.90 ± 0.04 | 13.00 ± 0.03 | 24.41 ± 0.07 | 11.73 ± 0.04 | 15.27 ± 0.07 |
|  | Red. χ^2^ | 1.31 | | | | |

**Supplementary Table 4** Summary of exchange parameters obtained from fitting ^13^C *R*_1ρ_ data measured on slGT-CGC at pH 6.9 and 25 °C in 100 % D_2_O. State B corresponds to the tautomeric WC-like G•T mismatch. Hoogsteen blowback refers to the passive sensing of the Watson-Crick to Hoogsteen exchange of the G6-C14 bp (state C) by the atoms of the G•T mismatch. Red. ${}^{2}$ denotes the reduced ${}^{2}$ obtained on fitting the *R*_1ρ_ data

| Parameter | G15-N1 | T5-N3 |
| --- | --- | --- |
| *p*_B_ (%) | 0.120 ± 0.001 | |
| *k*_exAB_ (s^-1^) | 6444 ± 122 | |
| Δω_AB_ (ppm) | 38.03 ± 0.19 | 17.33 ± 0.18 |
| *R*_1_ (s^-1^) | 3.07 ± 0.02 | 2.82 ± 0.01 |
| *R*_2_ (s^-1^) | 5.63 ± 0.09 | 5.60 ± 0.06 |
| Red. χ^2^ | 0.36 | |

**Supplementary Table 5** Summary of exchange parameters obtained from fitting ^15^N *R*_1ρ_ data measured on slGT-GGC at pH 6.9 and 25 °C in 90 % H_2_O:10 % D_2_O. The ^13^C *R*_1ρ_ profiles for G15-C8 and T5-C6 (Supplementary Figure 9b) do not show RD, and hence were not fit to extract exchange parameters. State B corresponds to the tautomeric WC-like G•T mismatch. Red. ${}^{2}$ denotes the reduced ${}^{2}$ obtained on fitting the *R*_1ρ_ data

| Parameter | T5-C3' |
| --- | --- |
| *p*_B_ (%) | 0.112 ± 0.007 |
| *k*_exAB_ (s^-1^) | 1657 ± 189 |
| Δω_AB_ (ppm) | 3.01 ± 0.13 |
| *R*_1_ (s^-1^) | 1.65 ± 0.02 |
| *R*_2_ (s^-1^) | 13.29 ± 0.07 |
| Red. χ^2^ | 0.55 |

**Supplementary Table 6** Summary of exchange parameters obtained from fitting ^13^C *R*_1ρ_ data measured on slGT-GGC at pH 6.9 and 25 °C in 100 % D_2_O. The ^13^C *R*_1ρ_ profiles for G15-C3', T5-C4', G15-C4', T5-C6 and G15-C8 (Figure 5b) do not show relaxation dispersion, and hence were not fit to extract exchange parameters. State B corresponds to the tautomeric WC-like G•T mismatch. Red. ${}^{2}$ denotes the reduced ${}^{2}$ obtained on fitting the *R*_1ρ_ data

| Parameter | G15-N1 | T5-N3 | T5-C4' |
| --- | --- | --- | --- |
| *p*_B_ (%) | 0.065 ± 0.003 | | |
| *p_C_* (%) | 0.566 ± 0.089 | | |
| *k*_exAB_ (s^-1^) | 4029 ± 352 | | |
| *k*_exAC_ (s^-1^) | 1201 ± 199 | | |
| Δω_AB_ (ppm) | 38.33 ± 0.43 | 15.81 ± 1.01 | -0.04 ± 8.74 |
| Δω_AC_ (ppm) | 3.52 ± 0.27 | 53.51 ± 0.28 | 1.09 ± 0.11 |
| *R*_1_ (s^-1^) | 2.14 ± 0.02 | 2.26 ± 0.04 | 1.36 ± 0.04 |
| *R*_2_ (s^-1^) | 6.96 ± 0.10 | 5.94 ± 0.11 | 20.14 ± 0.18 |
| Red. χ^2^ | 0.75 | | |

**Supplementary Table 7** Summary of exchange parameters obtained from fitting ^13^C and ^15^N *R*_1ρ_ data measured on slGT-GGC at pH 8.8 and 10 °C in 90 % H_2_O:10 % H_2_O. State B corresponds to the tautomeric WC-like G•T mismatch while state C corresponds to the anionic G•T**^¯^** mismatch. Red. ${}^{2}$ denotes the reduced ${}^{2}$ obtained on fitting the *R*_1ρ_ data

**References**

1. Trott, O. & Palmer, A.G., 3rd. Theoretical study of R(1rho) rotating-frame and R2 free-precession relaxation in the presence of n-site chemical exchange. *J Magn Reson* **170**, 104-12 (2004).

2. Rangadurai, A., Szymaski, E.S., Kimsey, I.J., Shi, H. & Al-Hashimi, H.M. Characterizing micro-to-millisecond chemical exchange in nucleic acids using off-resonance R1rho relaxation dispersion. *Prog Nucl Magn Reson Spectrosc* **112-113**, 55-102 (2019).

3. Bothe, J.R., Stein, Z.W. & Al-Hashimi, H.M. Evaluating the uncertainty in exchange parameters determined from off-resonance R1rho relaxation dispersion for systems in fast exchange. *J Magn Reson* **244**, 18-29 (2014).

4. Nomura, K. et al. DFT Calculations on the Effect of Solvation on the Tautomeric Reactions for Wobble Gua-Thy and Canonical Gua-Cyt Base-Pairs. *J Mod Phys* **4**, 422-431 (2013).

5. Brovarets, O.O. & Hovorun, D.M. How many tautomerization pathways connect Watson-Crick-like G*.T DNA base mispair and wobble mismatches? *J Biomol Struct Dyn* **33**, 2297-315 (2015).

6. Wagenmakers, E.J. & Farrel, S. AIC model selection uisng Akaike weights. *Psychon Bull Rev* **11**, 192-196 (2004).

7. Burnham, K.P. & Anderson, D.R. Multimodel inference: Understanding AIC and BIC in model selection. *Socio. Meth. Res.* **33**, 261-304 (2004).
